# Natural malaria infection elicits rare but potent neutralizing antibodies to the blood-stage antigen RH5

**DOI:** 10.1016/j.cell.2024.06.037

**Published:** 2024-09-05

**Authors:** Lawrence T. Wang, Andrew J.R. Cooper, Brendan Farrell, Kazutoyo Miura, Ababacar Diouf, Nicole Müller-Sienerth, Cécile Crosnier, Lauren Purser, Payton J. Kirtley, Maciej Maciuszek, Jordan R. Barrett, Kirsty McHugh, Rodney Ogwang, Courtney Tucker, Shanping Li, Safiatou Doumbo, Didier Doumtabe, Chul-Woo Pyo, Jeff Skinner, Carolyn M. Nielsen, Sarah E. Silk, Kassoum Kayentao, Aissata Ongoiba, Ming Zhao, Doan C. Nguyen, F. Eun-Hyung Lee, Angela M. Minassian, Daniel E. Geraghty, Boubacar Traore, Robert A. Seder, Brandon K. Wilder, Peter D. Crompton, Gavin J. Wright, Carole A. Long, Simon J. Draper, Matthew K. Higgins, Joshua Tan

**Affiliations:** 1 Antibody Biology Unit, Laboratory of Immunogenetics, National Institute of Allergy and Infectious Diseases, National Institutes of Health, Rockville, MD 20852, USA; 2 Vaccine Research Center, National Institute of Allergy and Infectious Diseases, National Institutes of Health, Bethesda, MD 20892, USA; 3 Department of Biochemistry, University of Oxford, South Parks Road, Oxford OX1 3QU, UK; 4 Medical Scientist Training Program, University of California, San Diego School of Medicine, La Jolla, CA 92093, USA; 5 Kavli Institute for Nanoscience Discovery, University of Oxford, Dorothy Crowfoot Hodgkin Building, South Parks Road, Oxford OX1 3QU, UK; 6 Laboratory of Malaria and Vector Research, National Institute of Allergy and Infectious Diseases, National Institutes of Health, Rockville, MD 20852, USA; 7 Wellcome Sanger Institute, Cambridge CB10 1SA, UK; 8 Department of Biology, Hull York Medical School, York Biomedical Research Institute, University of York, Heslington, York YO10 5DD, UK; 9 Vaccine and Gene Therapy Institute, Oregon Health & Science University, Portland, OR 97006, USA; 10 Malaria Infection Biology and Immunity Section, Laboratory of Immunogenetics, National Institute of Allergy and Infectious Diseases, National Institutes of Health, Rockville, MD 20852, USA; 11 Mali International Center of Excellence in Research, University of Sciences, Technique and Technology of Bamako, Point G, BP 1805 Bamako, Mali; 12 Clinical Research Division, Fred Hutchinson Cancer Research Center, Seattle, WA 98109, USA; 13 Protein Chemistry Section, Research Technologies Branch, National Institute of Allergy and Infectious Diseases, National Institutes of Health, Rockville, MD 20852, USA; 14 Division of Pulmonary, Allergy, Critical Care, and Sleep Medicine, Emory University, Atlanta, GA 30322, USA; 15 NIHR Oxford Biomedical Research Centre, Oxford OX3 9DU, UK

**Keywords:** RH5, *Plasmodium falciparum*, monoclonal antibodies, malaria, natural infection, vaccine design

## Abstract

*Plasmodium falciparum* reticulocyte-binding protein homolog 5 (RH5) is the most advanced blood-stage malaria vaccine candidate and is being evaluated for efficacy in endemic regions, emphasizing the need to study the underlying antibody response to RH5 during natural infection, which could augment or counteract responses to vaccination. Here, we found that RH5-reactive B cells were rare, and circulating immunoglobulin G (IgG) responses to RH5 were short-lived in malaria-exposed Malian individuals, despite repeated infections over multiple years. RH5-specific monoclonal antibodies isolated from eight malaria-exposed individuals mostly targeted non-neutralizing epitopes, in contrast to antibodies isolated from five RH5-vaccinated, malaria-naive UK individuals. However, MAD8–151 and MAD8–502, isolated from two malaria-exposed Malian individuals, were among the most potent neutralizers out of 186 antibodies from both cohorts and targeted the same epitopes as the most potent vaccine-induced antibodies. These results suggest that natural malaria infection may boost RH5-vaccine-induced responses and provide a clear strategy for the development of next-generation RH5 vaccines.

## Highlights

• Malaria-exposed individuals have rare but detectable RH5-specific B cells
• Potency of RH5 mAbs from infection and vaccination is tightly linked to their epitope
• Infection can elicit very potent mAbs that target sites of vulnerability on RH5
• Malaria infection may boost protective RH5-vaccine-induced antibody responses

## Graphical abstract

**Figure:**
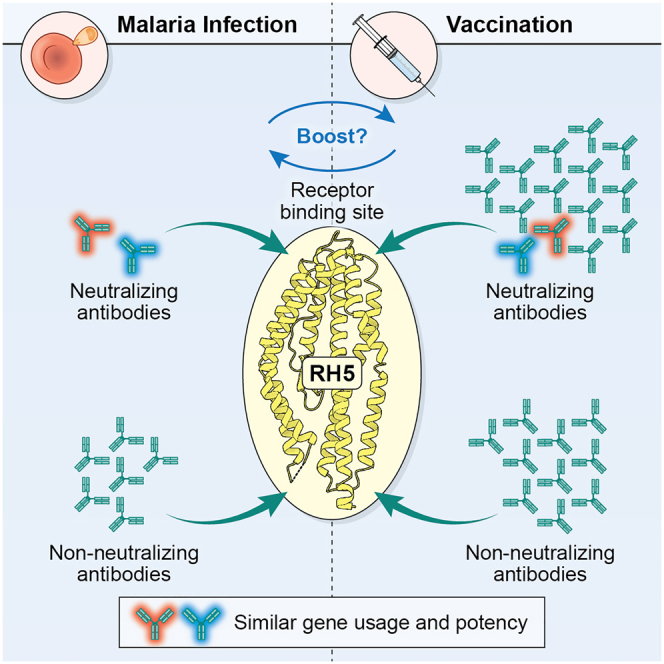

## Teaser

An in-depth analysis of the naturally acquired antibody response to the malaria vaccine candidate RH5 reveals that infection can induce rare but potently neutralizing antibodies that target the same sites on the protein as potent vaccine-induced antibodies.

## Introduction

Developing highly effective tools against *Plasmodium falciparum* (*P. falciparum*), the parasite that causes the most severe form of malaria, is a long-sought global health priority.^1^ Encouraging progress has been made recently in the development of vaccines and monoclonal antibodies (mAbs) that target *P. falciparum* sporozoites, the parasite stage transmitted by *Anopheles* mosquitoes to initiate infection in the human host.^2^^,^^3^^,^^4^^,^^5^ However, the protection provided by these interventions does not extend to the subsequent disease-causing blood stage of infection due to antigenic differences between the two parasite stages. Given the potential for only a single breakthrough sporozoite to cause malaria disease, there is a strong impetus to develop tools and strategies that target blood-stage parasites as a second line of defense. However, decades of effort to develop blood-stage vaccines have been hampered by the parasite’s ability to evade antibodies by mutating its surface antigens and invading erythrocytes via multiple redundant pathways.^6^^,^^7^

Blood-stage vaccine development was bolstered by the identification of a well-conserved complex used by *P. falciparum* merozoites to invade host erythrocytes. This complex was initially found to be composed of reticulocyte-binding protein homolog 5 (RH5), cysteine-rich protective antigen (CyRPA), and RH5-interacting protein (RIPR).^8^^,^^9^ A recent study identified two other binding partners in this complex: *Plasmodium* thrombospondin-related apical merozoite protein (PTRAMP) and cysteine-rich small secreted protein (CSS).^10^^,^^11^ Of all the members of this complex, RH5 is the most well-studied as a vaccine candidate and is at the most advanced stage of clinical development.^12^ RH5 plays an indispensable role in mediating merozoite invasion by binding the erythrocyte surface protein basigin.^13^^,^^14^^,^^15^ Furthermore, RH5 vaccination can elicit broadly neutralizing antibodies in animals^16^^,^^17^ and a single RH5-specific mAb conferred protection against blood-stage *P. falciparum* challenge in *Aotus* monkeys.^18^ Nevertheless, the biological role and location of RH5 during erythrocyte invasion presents unique challenges for vaccine development. RH5 is not constitutively expressed on the merozoite surface but is sequestered within intracellular organelles and is only released to the surface just prior to engagement of basigin, providing a limited time window for antibodies to bind. Therefore, antibodies targeting RH5 need to be present at sufficient concentrations and must bind quickly to prevent merozoite invasion. This challenge is illustrated by findings from a recent phase I/II clinical trial in which malaria-naive individuals were vaccinated with a recombinant RH5 vaccine and subsequently exposed to controlled human malaria infection (CHMI).^19^ This trial reported that the vaccine significantly delayed blood-stage growth of parasites but did not elicit sufficient neutralizing antibodies to prevent malaria infection.^19^ Therefore, more work is needed to improve the ability of RH5 vaccines to elicit both higher antibody titers and a more focused response toward regions of the protein that prevent merozoite invasion of erythrocytes.

A key recent development in the RH5 vaccine field is the initiation of clinical trials in sub-Saharan Africa to test RH5 vaccine safety, immunogenicity, and efficacy against clinical malaria in endemic regions.^12^ This development is essential, given that the RH5 vaccine is being developed primarily for use in these regions. However, an additional factor to consider is that final deployment of the vaccine would involve immunization of individuals who have experienced prior malaria episodes and will likely experience future episodes during and after RH5 vaccination. These naturally acquired antibody responses would be intertwined with those from vaccination, potentially boosting or interfering with the vaccine-induced antibody response and altering the degree of protection against malaria. Therefore, the design of an RH5 vaccine should consider not just RH5-specific antibody responses from malaria-naive, vaccinated individuals but also responses from individuals who have experienced malaria infection. Currently, the longevity and function of anti-RH5 antibodies and the frequency of RH5-reactive B cells elicited by natural infection remain poorly understood, aside from reports that anti-RH5 polyclonal antibody titers in malaria-exposed humans are low^16^^,^^20^^,^^21^^,^^22^ but correlate with protection against malaria.^21^^,^^23^^,^^24^^,^^25^ More generally, much remains to be elucidated about the molecular details of the human antibody response to RH5. To our knowledge, only a single study on human RH5-specific mAbs has been reported, in which 17 mAbs were isolated from malaria-naive, RH5-vaccinated individuals and characterized in detail.^26^ Furthermore, no such studies of RH5-specific mAbs from naturally infected individuals have been performed.

Here, we investigated the B cell response to RH5 during natural malaria infection and compared this to the response elicited by RH5 vaccination. We found evidence of a weak response to RH5 despite repeated malaria infections over multiple years, both at the level of RH5-reactive B cells and circulating polyclonal anti-RH5 antibodies. We analyzed a panel of 186 RH5-specific mAbs derived from natural infection and RH5 vaccination. Neutralization potency was strongly associated with binding to three distinct regions of RH5 proximal to the receptor-binding site that contacts basigin. Although mAbs induced by malaria infection were on average less potent than mAbs derived from vaccination, two infection-derived mAbs (MAD8–151 and MAD8–502) targeted critical RH5 epitopes and were among the most potently neutralizing mAbs in the entire panel. Moreover, we found evidence for shared V gene usage between infection- and vaccination-derived mAbs. In particular, infection-derived MAD8–151 and vaccination-derived MAD10–255 targeted the same epitope with similar potency and shared identical heavy and light chain V(D)J gene usage and CDR1-3 lengths, providing evidence for convergent B cell selection despite differences in the type of exposure to RH5 and donor geographic origin. These findings suggest that natural infection is capable of inducing neutralizing antibody responses that could boost, and be boosted by vaccination, providing valuable insight for fine-tuning the design of next-generation RH5 vaccines.

## Results

### Malaria exposure elicits weak B cell responses to RH5 despite repeated infection

**Figure 1:**
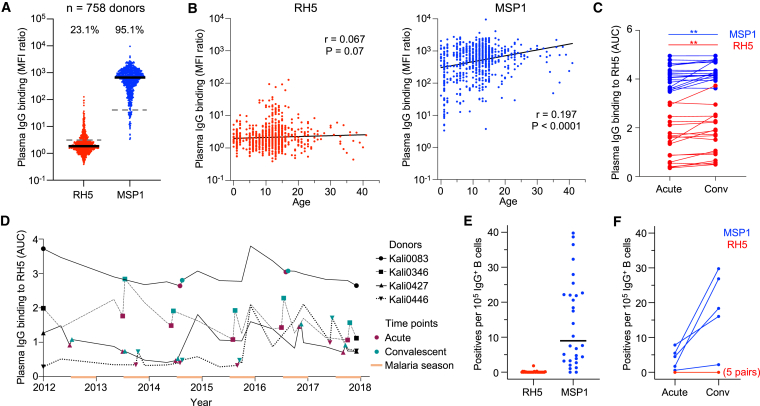
Malaria-exposed individuals have weak B cell responses to RH5 despite repeated infection (A) IgG binding to RH5 and MSP1 of plasma from individuals living in Kalifabougou, Mali (*n* = 758 donors). Median fluorescence intensity (MFI) values shown are after division with values for the negative control antigen, CD4. Bars show median values. Dashed lines show average IgG binding of plasma from 42 US donors. The numbers above each group show the percentage of individuals with plasma binding above this negative control average. (B) Cross-sectional analysis of relationship between plasma IgG binding to RH5 and MSP1 with age. Correlation r and *p* values were determined using Spearman correlation. (C) Plasma IgG binding to RH5 of samples from paired acute and convalescent time points. Acute samples were collected upon diagnosis of clinical malaria and convalescent samples were collected ∼1 week after the acute samples. The Wilcoxon sign-rank test was used to analyze changes in binding between the two time points. ∗∗*p* < 0.01. (D) RH5-specific IgG levels in plasma of four Malian donors from whom we isolated RH5-specific mAbs over a period of 6 years. The orange bars denote the malaria season, which occurs in the latter half of each year. (E) Frequency of RH5- or MSP1-positive wells of cultured IgG^+^ B cells from 30 analyzed donors. Two runs where B cells from different donors were pooled into the same wells were excluded from this analysis. Bars show median values. (F) Frequency of RH5- or MSP1-positive wells of cultured IgG^+^ B cells from five pairs of acute and convalescent samples. Two pairs are from the same donor (Kali0346) but were taken from different malaria seasons. For RH5, the five pairs are completely overlapping. See also Figure S1.

To investigate the antibody response to RH5 during natural infection, we studied a longitudinal cohort of 758 individuals (1 month–41 years of age) living in Kalifabougou, Mali.^27^ This rural community experiences intense seasonal malaria between July and December, during which the estimated entomological inoculation rate is 2 infective bites per person per day at the peak of the season. Annual collection of samples before and after the malaria season, as well as during active and passive surveillance for clinical malaria cases, allowed us to study antibody levels to RH5 during critical time points. To analyze long-lasting antibody responses to *P. falciparum* antigens, we initially focused on plasma collected in May after the 5-month dry season when malaria transmission is minimal. RH5-specific immunoglobulin G (IgG) levels in plasma from all 758 donors were compared with IgG levels to merozoite surface protein-1 (MSP1), an immunodominant protein expressed during the same stage of the parasite life cycle.^21^^,^^28^ Polyclonal IgG binding was lower for RH5 compared with MSP1 (23.1% versus 95.1% of individuals with binding above background, respectively) (Figure 1A). We examined whether IgG responses to RH5 and MSP1 were associated with age in a cross-sectional survey (Figure 1B). MSP1-specific IgG levels increased with age, particularly in the first 5 years of life, consistent with rapid activation of B cells by an immunodominant blood-stage antigen. However, there was no association between age and RH5-specific IgG levels, suggesting that repeated exposure to merozoites over multiple malaria seasons did not trigger a long-lasting antibody response to this antigen. This could either be due to the inability of RH5 to activate B cells or the induction of only short-lived antibody-secreting B cells during infection. To distinguish between the two possibilities, we screened paired acute (collected on the day of malaria diagnosis) and convalescent (∼1 week after acute sample collection) plasma samples from donors who had documented cases of clinical malaria. Antibody levels to MSP1 increased in this 1-week window (*p* < 0.01), confirming that these samples captured a suitable time period to measure the induction of anti-merozoite responses (Figure 1C). Antibody levels to RH5 also increased in this time period (*p* < 0.01), indicating that natural infection triggers a detectable antibody response to RH5. Indeed, when we tracked the RH5-specific IgG profile of 4 individuals over a period of 6 years, we observed frequent occasions in which a documented case of clinical malaria resulted in a sharp increase in RH5 antibody levels (Figure 1D). However, these responses were often followed by a rapid decline in antibody levels, consistent with the induction of short-lived antibody responses against RH5.

Next, we investigated whether RH5-reactive IgG^+^ B cells are detectable in individuals exposed to repeated malaria infection. We selected peripheral blood mononuclear cells (PBMCs) from 30 individuals (3–19 years of age) whose RH5-specific circulating IgG levels were within the highest 10% of the Malian cohort. We activated IgG^+^ B cells from these donors polyclonally and screened their supernatants for reactivity to RH5 and MSP1. Out of 10.9 million IgG^+^ B cells in 4,307 wells (2,500 cells/well) from the 30 donors, we identified 1,475 MSP1^+^ wells but only 14 RH5^+^ wells (Figure 1E). To determine whether a documented clinical malaria episode triggers a detectable increase in RH5-reactive B cells, we analyzed IgG^+^ B cells from five paired acute and convalescent time points. We detected an increase in MSP1- but not RH5-reactive B cells (Figure 1F). Collectively, these findings suggest that RH5-reactive B cells, while present, are rare in malaria-exposed individuals and do not expand appreciably during acute malaria infection.

### Malaria exposure elicits rare but potent neutralizing antibodies targeting RH5

**Figure S1:**
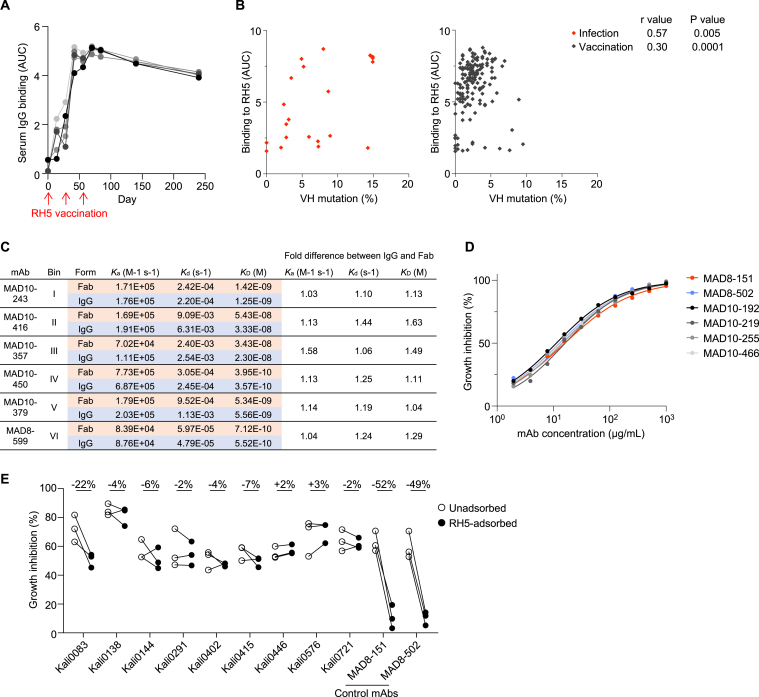
Infection- and vaccination-induced antibody responses to RH5, related to Figures 1 and 2 (A) Serum IgG reactivity to RH5 after three doses of the RH5/AS01 vaccine in five malaria-naive individuals. (B) Association between RH5 binding and VH mutations of RH5-specific mAbs from natural infection (red) and vaccination (black). *p* and r values were calculated based on Spearman correlation. (C) Binding kinetics of RH5-specific IgG (targeting bins I–VI) and corresponding Fabs to RH5. Equimolar binding arms of each form (4.2 nM Fab and 2.1 nM IgG) were compared. K_a_, association rate constant; K_d_, dissociation rate constant; K_D_, equilibrium dissociation constant. (D) Growth inhibition titration curves of the six most potent RH5-specific mAbs. Data are shown from a representative experiment out of *n* = 2–3 experiments. MAD8–151 and MAD8–502 were isolated from infected donors while MAD10–192, MAD10–219, MAD10–255, and MAD10–466 were isolated from vaccinated donors. (E) Growth inhibition mediated by polyclonal IgG from naturally infected donors in an antigen-reversal assay, where samples are tested for activity with and without adsorption of RH5-specific antibodies with soluble antigen. Each pair of points represents an independent experiment. MAD8–151 and MAD8–502 are control RH5-specific mAbs. Kali0083 is the source donor of MAD8–502 and Kali0446 is the source donor of MAD8–151. The percentages at the top of the figure refer to the mean difference between the RH5-adsorbed and -unadsorbed inhibition values.

**Figure 2:**
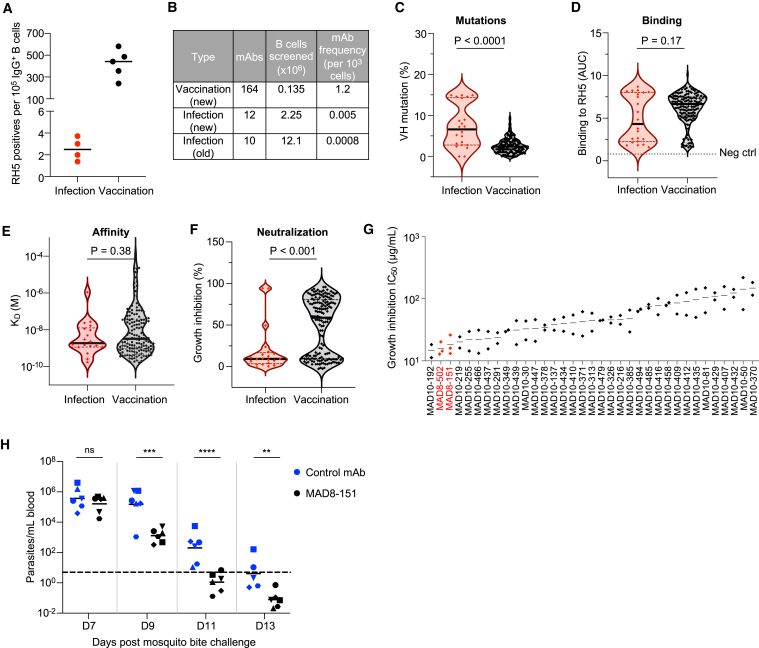
Malaria exposure elicits rare but potent neutralizing antibodies targeting RH5 (A) Frequency of RH5-positive wells of cultured IgG^+^ B cells from infected versus vaccinated donors. Bars show median values. (B) Number of mAbs isolated from infected versus vaccinated donors. The “new” method refers to the more sensitive but labor-intensive B cell screening approach while the “old” method refers to the original approach used to investigate B cells in Figure 1. (C) Heavy chain variable (VH) mutation frequencies of mAbs isolated from infected versus vaccinated donors. Bars show median values and dashed lines show quartiles. *p* value was calculated using the Mann-Whitney U test. (D) RH5 binding of mAbs isolated from infected versus vaccinated donors. Bars show median values and dashed lines show quartiles. The dotted line shows the binding of a negative control mAb, VRC01-LS. *p* value was calculated using the Mann-Whitney U test. (E) Binding affinity of mAbs isolated from infected versus vaccinated donors, as measured by the equilibrium dissociation constant (K_D_). Bars show median values and dashed lines show quartiles. *p* value was calculated using the Mann-Whitney U test. Stars indicate mAbs with a dissociation rate at the limit of detection (1 × 10^−5^ s^−1^), which was the value used to calculate the K_D_ in this graph. Data were obtained for 18/22 infection-derived mAbs and 151/164 vaccination-derived mAbs; the remaining mAbs had insufficient binding signal or were acid-denatured during the workflow. (F) Growth inhibition of mAbs isolated from infected versus vaccinated donors. All mAbs were tested at 1 mg/mL. MAD8–486 and MAD10–44 are not shown due to poor expression yields, which precluded testing at a 1 mg/mL concentration. Bars show median values and dashed lines show quartiles. *p* value was calculated using the Mann-Whitney U test. (G) Growth inhibition half maximal inhibitory concentration (IC_50_) values from titrations of the 35 most potent mAbs. Each point represents an independent experiment. Bars show the mean value. (H) Parasitemia in FRG HuHep mice challenged by infectious mosquito bite (carrying *P. falciparum* NF54 sporozoites) and administered 625 μg/mL of either MAD8–151 or a negative control mAb 1245 (Scally et al.^30^). The dashed line shows the limit of detection of parasite quantification by reverse-transcriptase quantitative PCR (RT-qPCR). Each symbol denotes an individual mouse. Bars show geometric mean values. One mouse in the control group was euthanized after day 11 due to poor health. Differences between mouse groups were analyzed by mixed-effect analysis with Šídák’s multiple comparisons test. ns, non-significant. ^∗^*p* < 0.05, ^∗∗^*p* < 0.01, ^∗∗∗^*p* < 0.001, ^∗∗∗∗^*p* < 0.0001. See also Figure S1.

Apart from quantifying the antibody response to RH5, we were interested in assessing the quality of the response by isolating RH5-specific mAbs from malaria-exposed individuals. To increase the probability of identifying rare RH5-reactive B cell clones, we used a more labor-intensive but sensitive approach for mAb screening that utilized sequential 384-well oligoclonal and optofluidic monoclonal B cell culture.^29^ Four individuals with high RH5-specific polyclonal IgG (within the highest 10% of the Malian cohort), and who had multiple PBMC samples available, were selected for mAb isolation using this approach. To compare antibodies elicited by natural infection and vaccination, mAbs were also isolated in parallel from five malaria-naive UK adults who had been vaccinated with recombinant RH5 formulated with the adjuvant AS01_B_.^19^ In these individuals, RH5 vaccination induced long-lasting polyclonal IgG antibodies that only declined slightly 6 months after the final vaccine dose (Figure S1A). We detected >100-fold higher frequencies of RH5-reactive IgG^+^ B cells in vaccinated versus malaria-exposed individuals (Figure 2A). This difference in B cell frequency was further emphasized by the vastly different number of RH5-specific mAbs we isolated from each group of individuals. From the vaccinees, 164 RH5-specific mAbs were isolated from 0.135 million IgG^+^ B cells, whereas, from the malaria-exposed cohort, only 22 RH5-specific mAbs were isolated from 14.4 million IgG^+^ B cells, including B cells from the initial screens described in the previous section (Figure 2B). The mAbs from malaria-exposed donors had acquired more somatic mutations than those from the vaccinated donors, consistent with repeated B cell exposures to RH5 over an extended period of time (Figure 2C). Surprisingly, this did not translate to stronger binding to RH5, based on area under the curve (AUC) measurements of binding to RH5-coated beads (Figure 2D). Upon closer examination, we found that the number of mutations was associated with improved binding to RH5 in both cohorts when they were examined separately (Figure S1B). However, a few highly mutated mAbs in the infection group elevated the average number of mutations in this group (Figure S1B). We also examined the affinity of binding of the mAbs to RH5 by surface plasmon resonance (SPR). To evaluate 1:1 binding interactions and avoid avidity effects, we immobilized the mAb panel on the chip surface and measured association and dissociation to soluble RH5. We confirmed with a smaller number of mAbs that this approach yielded similar results to fragment antigen binding (Fab) in a head-to-head comparison (Figure S1C). We successfully obtained affinity data on most of the mAbs and found no differences in affinity between the infection- and vaccine-derived mAbs (Figure 2E).

The panel of 186 RH5-specific mAbs was assessed for function using the growth inhibition assay (GIA), a key *in vitro* reference assay that measures neutralizing activity against blood-stage *P. falciparum* and is highly correlated with *in vivo* protection mediated by RH5 antibodies.^18^^,^^19^^,^^31^ Surprisingly, most of the mAbs from natural infection were non-neutralizing, with only 2 (MAD8–151 and MAD8–502) out of 21 mAbs (9.5%) surpassing the 50% inhibition threshold when tested at 1 mg/mL and a third mAb (MAD8–501) falling just under this threshold (Figure 2F). In contrast, 93 out of 163 vaccine-derived mAbs (57.1%) surpassed this threshold. However, titrations of the 35 mAbs with the highest inhibition values at 1 mg/mL revealed that MAD8–151 and MAD8–502 were among the most potently neutralizing mAbs, even when compared with the best vaccination-derived mAbs (Figures 2G and S1D), suggesting that natural infection can elicit potent, albeit uncommon, RH5-specific mAbs.

To determine whether circulating RH5-specific antibodies in infected donors reach sufficient levels to have a detectable functional effect, we screened polyclonal IgG from the source donors of MAD8–151 and MAD8–502, as well as several other Malian donors with high RH5 reactivity, in an antigen-reversal GIA.^32^ In this assay, growth inhibition is compared with and without the adsorption of RH5-specific antibodies to take only RH5-specific antibody activity into account. Only the source donor of MAD8–502, Kali0083, showed a consistent reduction in neutralization when RH5-specific antibodies were adsorbed (Figure S1E), indicating that RH5-specific antibodies rarely reach levels that enable observation of function at the polyclonal level.

We assessed the *in vivo* potency of MAD8–151 against *P. falciparum* transmitted by mosquito bite using a previously established humanized mouse model with modifications.^33^ In this *in vivo* model, blood-stage *P. falciparum* merozoites that emerge from the liver are permitted to persist in the blood for a restricted period of time through repeated infusion of human erythrocytes. In mice administered the isotype control mAb (mAb 1245),^30^ the geometric mean parasitemia was 3.7 × 10^6^ parasites/mL on day 7, maintained at a slightly lower level (1.5 × 10^6^ parasites/mL) on day 9, and slowly declined through day 13, with 6/6 mice and 2/5 mice remaining above the limit of detection (5 parasites/mL) on days 11 and 13, respectively (Figure 2H). This gradual reduction in blood stage parasitemia between days 9 to 13 post-sporozoite infection is likely due to clearance of human red blood cells and is consistent with previous^33^ and more recent work on a similar Fah^−^^/^^−^/Rag2^−^^/^^−^/Il2rg^−^^/^^−^ (FRG) HuHep infection model.^34^ In contrast, mice administered the infection-derived RH5-specific mAb MAD8–151 cleared parasitemia more rapidly (*p* < 0.01 on days 9, 11, and 13). There was clearly reduced parasitemia in these mice on day 9 (geometric mean parasitemia 118-fold lower than in control mice), five out of six mice were parasite-negative by day 11 and all mice were parasite-negative by day 13. These results suggest that infection-derived RH5-specific mAbs can function to control *P. falciparum* parasitemia *in vivo* following mosquito bite challenge.

### mAbs induced by natural infection primarily target non-neutralizing RH5 epitopes

**Figure S2:**
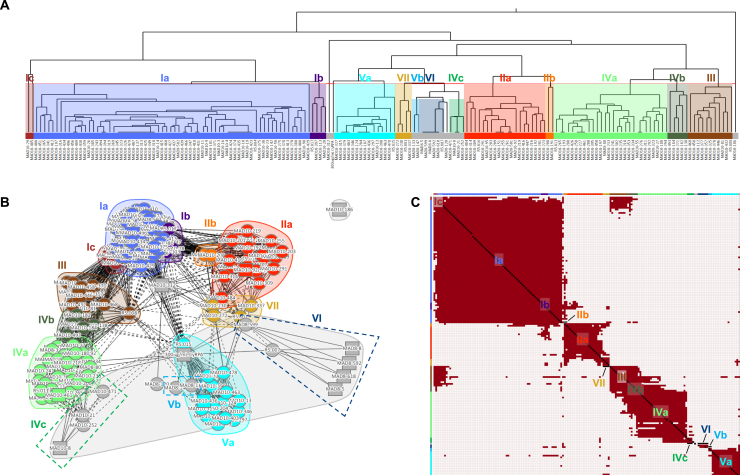
Detailed epitope binning analysis based on mAb competition profiles, related to Figure 3 (A) Hierarchical clustering of mAbs using the McQuitty method (Epitope software), based on SPR-based mAb competition. Epitope bins were derived from mAb clustering and competition profiles and are shown in different colors. Bins Ia–Vb were identified based on the hierarchical tree, with two mAbs in Ic clustered into a separate bin due to limited overlap with group Ia mAbs and extensive competition with group III mAbs (see Figure S2C, top left). Bin VI was identified based on competition with the anchor mAb R5.007, which targets the RH5 intrinsic loop. (B) Epitope bins of RH5-specific mAbs shown in a two-dimensional (2D) representation, clustered based on competition profiles. Solid lines indicate two-way competition, dashed lines indicate one-way competition. Circular nodes represent mAbs analyzed as both ligand and analyte; square nodes represent mAbs analyzed as ligand or analyte only. The layout.auto display (Epitope software) was used to generate this plot. (C) Heatmap showing competition of mAbs targeting RH5. Red squares signify competition between the corresponding analyte and ligand mAb, while cream squares indicate no competition. The colors at the side and top are those of the 13 individual bins. 13/22 infection-derived mAbs and 153/164 vaccine-derived mAbs were mappable in this assay; the remaining mAbs were incompatible with the workflow, e.g., due to acid sensitivity during the regeneration step.

**Figure 3:**
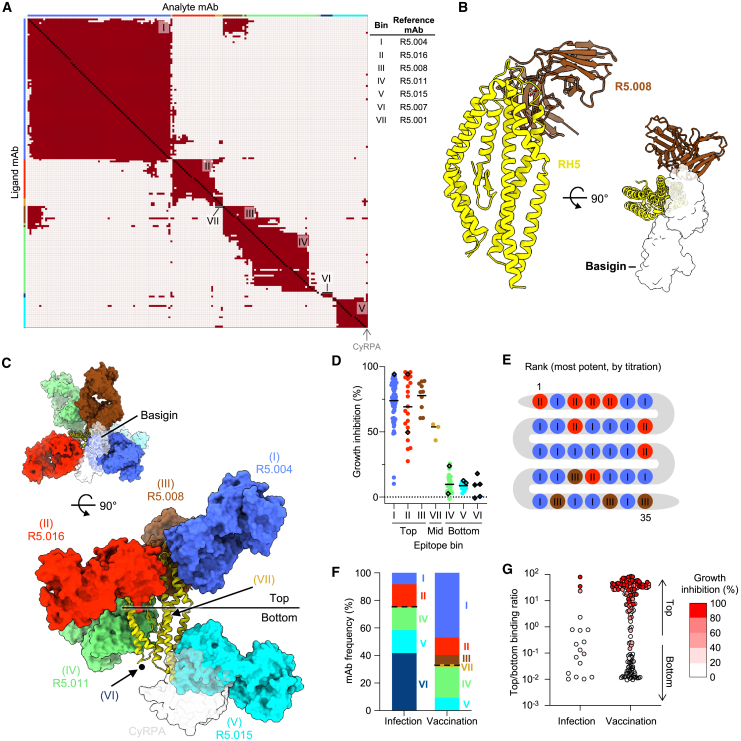
mAbs from natural infection primarily target non-neutralizing RH5 epitopes (A) Heatmap showing competition of mAbs targeting RH5. Red squares signify competition between the corresponding analyte and ligand mAb, while cream squares indicate no competition. The colors at the edges are those of the combined bins I–VII (e.g., bin I is formed from Ia–Ic). 13/22 infection-derived mAbs and 153/164 vaccine-derived mAbs were mappable in this assay; the remaining mAbs were incompatible with the workflow, e.g., due to acid sensitivity. (B) Crystal structure of RH5 (yellow) bound to the scFv of R5.008 (brown), each shown in cartoon. R5.008 competes with the binding of basigin (transparent white surface, PDB: 4U0Q) to RH5. (C) Neutralizing and non-neutralizing antibodies target the top and bottom of RH5, respectively. Representative antibody structures for each bin (I = R5.004, PDB: 6RCU; II = R5.016, PDB: 6RCV; III = R5.008; IV = R5.011, PDB: 6RCV; V = R5.015, PDB: 7PHU) are shown in surface representation on RH5 in cartoon. R5.008 is modeled as a Fab fragment for illustrative purposes. The binding locations of basigin (PDB: 4U0Q) and CyRPA (PDB: 8CDD) are shown as transparent surfaces in the top and side views, respectively, and the black dot indicates the location of the internal disordered loop of RH5. The approximate locations of bin VI (based on binding to the intrinsic loop) and bin VII (based on partial overlap with bins II and V) are shown by arrows. (D) Growth inhibition of mAbs, subdivided by major epitope bin. Bars show mean values. Infection-derived mAbs are shown as diamonds. (E) Epitope bins of 35 most potent RH5-specific mAbs, as determined by GIA titration. (F) Frequency of infection- and vaccination-derived mAbs in each RH5 epitope bin. The dotted lines separate bins at the top and bottom of RH5. (G) mAbs scored by binding proximity to the top of RH5, based on binding to RH5 with bins I–III pre-blocked versus bins IV–VI pre-blocked. Data points are geometric means from two independent experiments. Points are color-coded by GIA score at 1 mg/mL. See also Figures S2–S4.

As the differences in neutralization potency between the vaccination- and infection-derived mAbs could not be explained based on binding strength, we performed an SPR-based epitope binning assay to analyze the epitope specificity of mAbs from the two groups. We included reference RH5-specific mAbs with known binding sites to anchor epitope bins to specific regions of RH5.^26^ Using this approach, we mapped the binding sites of 13/22 infection-derived mAbs and 153/164 vaccine-derived mAbs. Based on the global competition profiles of the 166 mAbs, we identified 13 unique epitope bins on the RH5 surface (Figure S2A). Four bins were anchored by reference mAbs with known binding sites based on previously solved crystal structures: Ia (R5.004), IIa (R5.016), IVa (R5.011), and Va (R5.015).^26^ A fifth bin (VI) was anchored by a reference mAb (R5.007) that has been shown by peptide mapping to bind to the intrinsic disordered loop at the base of RH5.^26^ Three other bins contained reference mAbs with no prior structural or peptide mapping data, but with data from competition assays with RH5 binding partners to identify their general binding region: Ib (R5.017, competes with the RH5 receptor basigin), III (R5.008, competes with basigin), and VII (R5.001, competes with CyRPA).^26^ The remaining five bins were newly identified, with detailed competition data that allowed us to infer their location on the RH5 surface (prototypical mAbs in parentheses): Ic (MAD10–485, between locations Ia and III), IIb (MAD10–208, between Ib, IIa, and VII), IVb (MAD10–182, between III and IVa), IVc (MAD10–252, proximal to IVa but distant from IVb and III), and Vb (MAD8–133, partial overlap with Va and CyRPA, distant from all other bins) (Figures S2B and S2C). Based on similarities in the mAbs’ competition profiles, these 13 bins could be clustered into 7 major epitope bins, with three bins (I–III) located near the basigin-binding site (i.e., the "top" of RH5) and three bins (IV–VI) located at the opposite end of RH5 proximal to the binding site of CyRPA (i.e., the “bottom” of RH5) (Figure 3A). The 7^th^ bin (VII) contained mAbs that competed with both top (bin II) and bottom (bin V) mAbs and was classified as a “middle” bin (Figures 3A and 3C).

All of the major neutralizing RH5 epitopes have been characterized in detail through crystal structures with reference mAbs^26^^,^^35^ except bin III, a neutralizing site for which there is a lack of structural information.^26^ We therefore generated a single-chain variable fragment (scFv) for R5.008 and determined the crystal structure of this mAb bound to RH5 (Figure 3B; Table S1). We find that R5.008 binds to the top half of RH5 to an epitope predominantly formed from the C-terminal two helices (6 and 7) of the RH5-fold, together with the linking loop and the loop between helices 3 and 4. This epitope partly overlaps the binding site for the N-terminal domain of basigin,^14^ supporting previous observations that R5.008 directly blocks basigin binding.^26^ Comparison of the R5.008-bound structure with structures of RH5 bound to epitope I (R5.004) and epitope II (R5.016) shows that these three epitopes cover much of the top half of RH5 (Figure 3C). Each of these three epitope bins acts by blocking the binding of RH5 to basigin, either through direct competition as for R5.004 and R5.008 (Figures 3B and 3C),^26^ or by blocking the binding of RH5 to basigin-containing membrane protein complexes, as for R5.016.^36^ In contrast, epitope V overlaps directly with the CyRPA-binding site,^35^ while bin IV is located toward the side at the bottom half of RH5 and contains antibodies that potentiate the effect of neutralizing antibodies (Figure 3C).^26^

**Figure S3:**
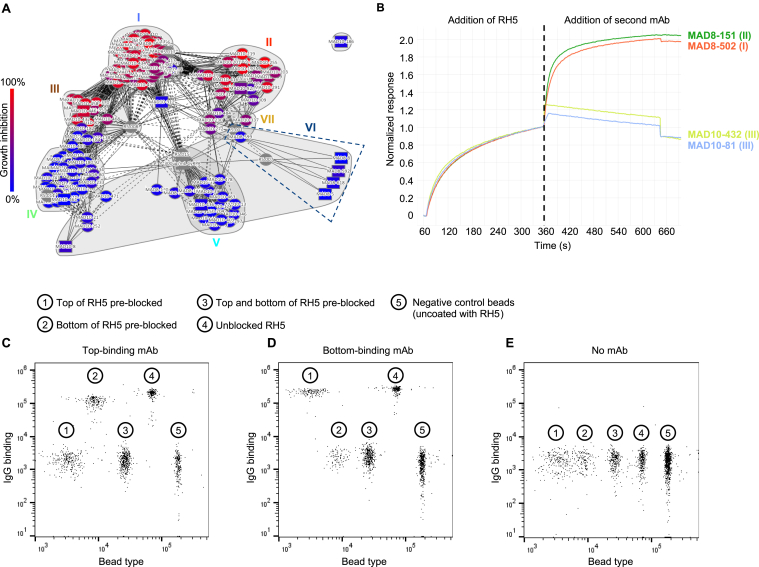
Discrete RH5 epitope communities correlate with antibody GIA score, related to Figure 3 (A) GIA-coded epitope bins of RH5-specific mAbs. Nodes are color-coded by GIA percentage at 1 mg/mL. Negative scores were set to zero. Reference mAbs and the antigen CyRPA, which were not analyzed by GIA, are colored gray. Solid lines indicate two-way competition, dashed lines indicate one-way competition. Circular nodes represent mAbs analyzed as both ligand and analyte; square nodes represent mAbs analyzed as ligand or analyte only. (B) SPR sensorgram showing binding of RH5 to bin IV mAb MAD8–323 on the chip surface, followed by binding of bin I, II, and III mAbs to the bound RH5. (C–E) Representative FACS plots from a multiplex bead-based assay to determine epitope localization of RH5-specific mAbs. Bead populations 1–3 display RH5 that was pre-blocked at top epitopes, bottom epitopes, or both. Bead population 4 displays unblocked RH5. Bead population 5 was not coated with RH5. Fluorescently labeled anti-IgG secondary antibody was used to determine the level of binding of each mAb. Results from a representative top-binding mAb (C), bottom-binding mAb (D), and no mAb negative control (E) are shown.

Strikingly, for both infection- and vaccination-derived mAbs, those targeting the top of RH5 (bins I–III) were predominantly neutralizing, those targeting the middle (bin VII) were partially neutralizing, while all mAbs targeting the bottom (bins IV–VI) were non-neutralizing (Figures 3D and S3A). Of the 35 most potent mAbs based on the GIA titration (Figure 2G), the top 25 mAbs bound to bins I and II, highlighting these two regions as the most effective RH5 target sites (Figure 3E). Given the partially overlapping competition profile between the neutralizing bin III and non-neutralizing bin IV mAbs (Figure 3A), we more closely examined the interactions between these bins using several mAbs as an example. We found that the infection-derived bin IV mAb MAD8–323 prevented the binding of vaccine-derived bin III mAbs MAD10–432 and MAD10–81 to RH5, but did not inhibit the binding of infection-derived bin I and II mAbs MAD8–151 and MAD8–502 (Figure S3B). Based on the binning analysis, only a minority of infection-derived mAbs (3/13, 23.1%) targeted neutralizing epitopes at the top of RH5, while the majority of vaccination-derived mAbs targeted these sites (100/153, 65.4%), explaining the discrepancy in potency between the two mAb groups (Figure 3F). To allow the analysis of the epitope specificity of a greater number of infection-derived mAbs, we developed a more sensitive competition assay in which RH5-coated beads were pre-blocked with a cocktail of IgA-switched RH5-specific mAbs targeting the top, bottom, or both regions of RH5 (Figures S3C–S3E). Binding of each IgG mAb to blocked and unblocked beads was then compared by flow cytometry using a fluorescently labeled anti-IgG secondary antibody. Consistent with the SPR-based analysis, only 4/18 (22.2%) infection-derived mAbs in this expanded panel targeted the top of RH5, while 107/159 (67.3%) of vaccination-derived mAbs targeted this region (Figure 3G). Taken together, these results suggest that natural infection, unlike vaccination, elicits more B cells that target non-neutralizing epitopes on the bottom of RH5 away from the basigin-binding site.

**Figure S4:**
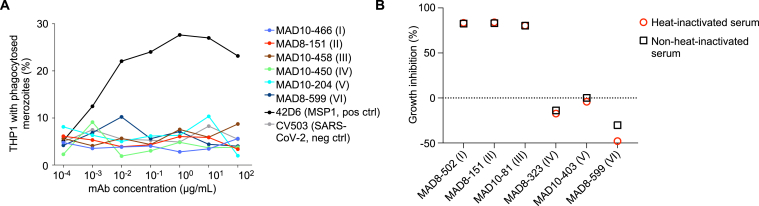
RH5-specific mAbs do not show Fc effector function in a phagocytosis- and complement-based assay, related to Figure 3 (A) Percentage of THP-1 monocyte cells with intracellular merozoites after opsonization of merozoites with various anti-RH5 mAbs. The anti-MSP1 mAb 42D6 (Patel et al.^62^) was used as a positive control. (B) Growth inhibition of 3D7 parasites with anti-RH5 mAbs in the presence of serum that was either non-heat inactivated or heat inactivated to eliminate complement activity. MAD8–502 and MAD8–151 were tested at 50 μg/mL, while the other 4 mAbs were tested at 200 μg/mL.

We investigated whether non-neutralizing RH5-specific mAbs could play a role in protection against malaria through anti-parasite Fc effector function. First, we tested the ability of mAbs in the major RH5 bins I–VI for their ability to opsonize merozoites for phagocytosis by THP-1 cells. None of the mAbs promoted phagocytosis, unlike a positive control MSP1-specific mAb (Figure S4A). The mAbs were also tested for their ability to inhibit parasite growth in the presence of complement, but the addition of complement did not enhance mAb activity for either top or bottom binders (Figure S4B).

### Binding of mAbs targeting bins I and II of RH5 strongly correlates with potency

**Figure 4:**
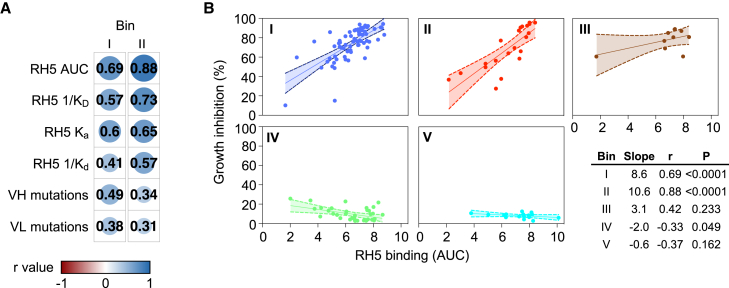
Binding strength of mAbs targeting bins I and II of RH5 is strongly correlated with neutralization potency (A) Spearman correlation between growth inhibition (% at 1 mg/mL) and several binding and sequence parameters for mAbs from bins I and II. The size of the circles and color intensity are proportional to the correlation r value. K_D_, equilibrium dissociation constant; K_a_, association rate constant; K_d_, dissociation rate constant. K_D_ and K_d_ were inverted to allow a direct comparison of positive correlations. Only mAbs from major bins I and II that had measurable values for all 6 parameters were included in this analysis. (B) Correlation between growth inhibition and RH5 binding based on AUC for mAbs, subdivided by bin. *p* and r values were derived from Spearman correlation. Bands show 95% confidence intervals. All mAbs from epitope bins (I–V) with measurable AUC values were considered in this analysis. Bins VI and VII were not included due to the few mAbs (≤5) in each bin.

The functional assessment of a large number of RH5-specific mAbs (*n* = 186) derived from vaccination and natural infection provided the opportunity to analyze this mAb panel for signatures of potent neutralization. As an initial test, we screened several binding and somatic mutation parameters for correlation with growth inhibition for mAbs from bins I and II, which showed the greatest potency (Figures 3E and 4A). The binding association rate was strongly associated with potency, which is consistent with previous findings on RH5-specific mAbs^26^ and reflects the need to quickly bind to RH5 to inhibit merozoite invasion, most likely due to transient exposure of RH5 during erythrocyte invasion. However, the magnitude of binding to RH5-coated beads (as measured by AUC), which captures antibody avidity and durability of the interaction, was most strongly correlated with potency, so we used this feature to analyze the wider mAb panel. Analysis of the mAbs after separation into the major epitope bins (I–V) revealed divergent patterns depending on the bin examined (Figure 4B). Binding to RH5 was strongly correlated with potency for mAbs in bins I and II. The correlation between binding strength and potency was less clear for bin III, but this bin contained relatively few mAbs (*n* = 10). For mAbs in bins IV and V, increasing binding strength did not increase potency, and even very strong binders in these groups were non-neutralizing. Importantly, the infection-derived mAbs followed the same pattern as the vaccination-derived mAbs, suggesting that similar rules govern the potency of both groups of mAbs. These results suggest that binding to specific epitopes at the top of RH5 is the primary determinant of mAb potency, regardless of the initial source of RH5 exposure.

### Potent RH5-specific mAbs use diverse VH genes and are clonally dispersed

**Figure 5:**
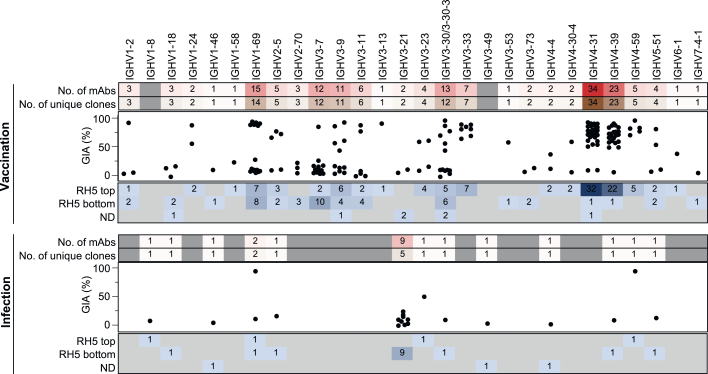
Potent RH5-specific mAbs use diverse VH genes and are clonally dispersed VH gene usage of mAbs derived from infection and vaccination. Numbers in the bottom panel represent the number of mAbs binding to the top or bottom of RH5 based on the bead competition assay. Bin VII middle binders, along with mAbs that could not be mapped, were classified as ND. MAD10–44 and MAD8–486 were excluded from the GIA plot as their expression levels were insufficient for testing at 1 mg/mL. See also Figure S5.

**Figure S5:**
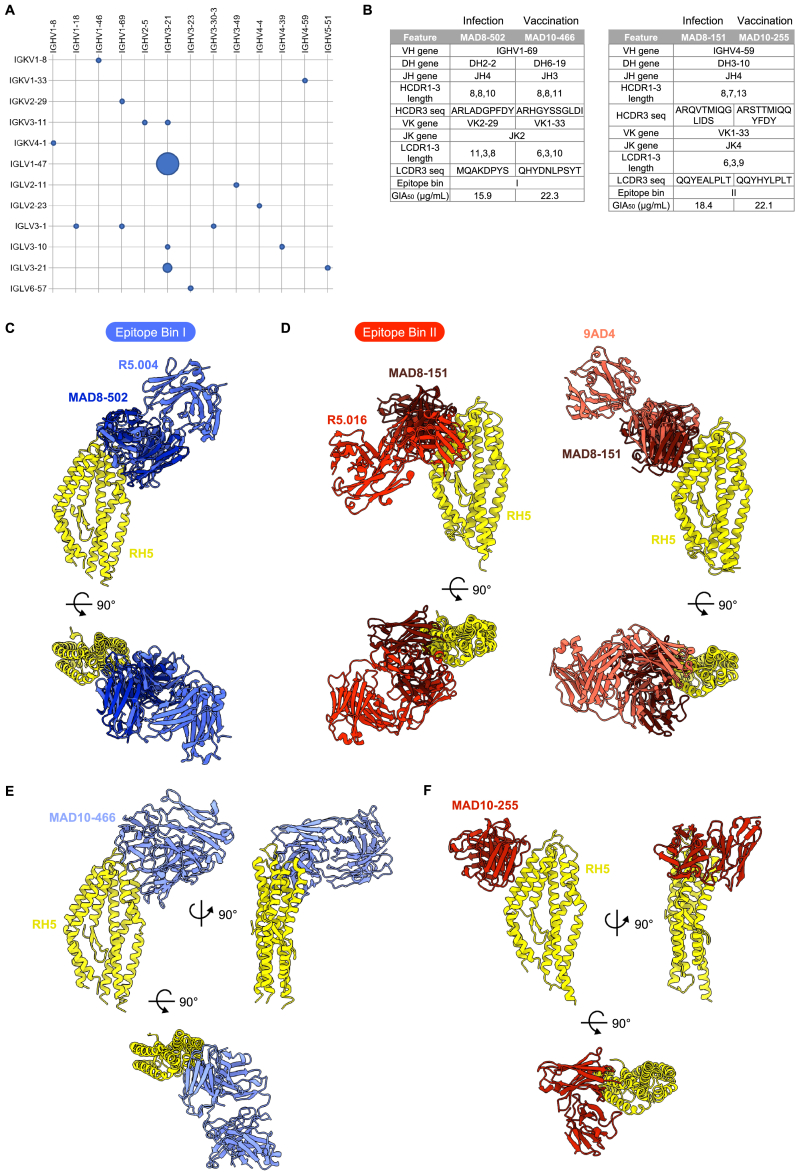
Genetic and structural features of RH5-specific antibodies from infection and vaccination, related to Figures 5 and 6 (A) Heavy (x axis) and light (y axis) chain V gene usage of RH5-specific mAbs from natural infection. The diameters of the circles are proportional to the number of mAbs using the corresponding heavy and light chain pair. The five mAbs using IGHV3-21/IGLV1-47 are clonally related, while the two mAbs using IGHV3-21/IGLV3-21 are not related and were isolated from different donors. (B) Genetic, binding, and functional features of MAD8–502 in comparison with MAD10–466, and MAD8–151 in comparison with MAD10–255. (C) Structural alignments of MAD8–502 scFv (dark blue) and R5.004 Fab (blue, PDB: 6RCU) bound to RH5 (yellow), showing that MAD8-502 belongs to epitope bin I. Complexes are shown as side and top views. (D) Structural alignments of MAD8–151 scFv (dark red) and R5.016 Fab (red, PDB: 6RCV) or 9AD4 Fab (pink, PDB: 4U0R) bound to RH5 (yellow), showing that MAD8-151 belongs to epitope bin II and shares an angle of approach to RH5 more similar to 9AD4 than R5.016. (E) Top and side views of the crystal structure of MAD10–466 Fab fragment (light blue) bound to RH5 (yellow). (F) Top and side views of the crystal structure of MAD10–255 scFv (red) bound to RH5 (yellow).

We compared the genetic backgrounds of mAbs induced by infection and vaccination and the relationship between heavy chain variable (VH) gene usage, target epitope, and potency. Vaccination-derived mAbs using diverse VH genes (e.g., IGHV1-2, IGHV1-24, IGHV1-69, IGHV3-7, IGHV3-9, IGHV3-11, IGHV3-13, IGHV3-30, IGHV3-33, IGHV4-31, IGHV4-39, and IGHV4-59) bound the top of RH5 and potently neutralized parasites, indicating a lack of conformational constraints on this target site that necessitate using specific antibody germlines to achieve binding (Figure 5). However, many of the same V genes were also used by mAbs targeting the bottom of RH5. Infection-derived mAbs used substantially fewer VH genes than those derived from vaccination, which was unsurprising given their smaller overall number (Figure 5). These mAbs showed a unique preference for IGHV3-21, with 9/22 mAbs all targeting the bottom of RH5 using this gene. The bias toward IGHV3-21 was not solely due to disproportionate expansion of a single clone, as the 9 mAbs originated from 5 distinct clonal lineages and were isolated from 4 different donors (Figure 5). We identified the most expanded lineage in the entire mAb panel here, with 5 mAbs targeting bin VI originating from a single ancestral B cell (Figure S5A). Strikingly, almost every vaccination-derived mAb (162 out of 164 mAbs), including those using common VH genes such as IGHV4-31 and IGHV4-39, originated from a distinct B cell ancestor, indicating clonal selection of a wide array of B cells in response to RH5 vaccination.

### Identification of a pair of doppelgangers from malaria infection and vaccination

**Figure 6:**
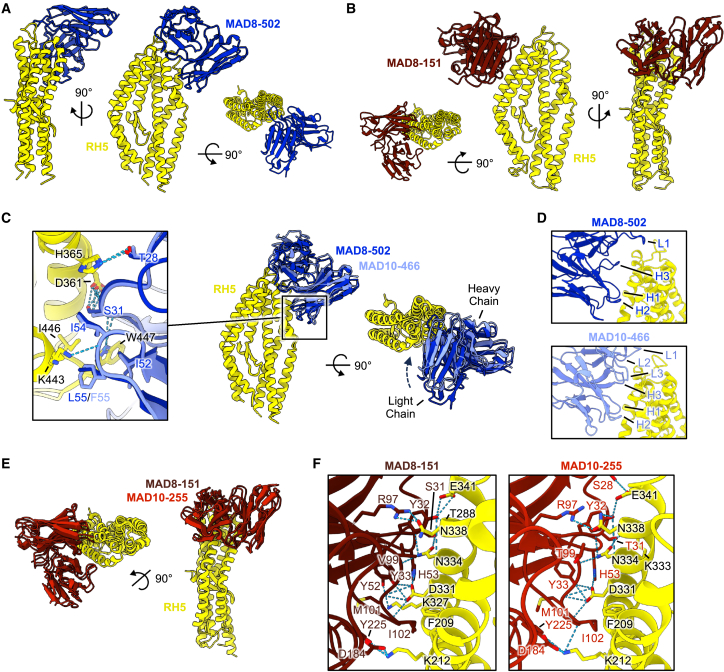
Crystal structures of naturally acquired RH5-targeting mAbs and vaccination-derived counterparts (A) Structure of MAD8–502 scFv (dark blue) bound to RH5 (yellow) from several views, shown in cartoon representation. (B) Structure of MAD8–151 scFv (dark red) bound to RH5 (yellow), as in (A). (C) Overlay of naturally acquired MAD8–502 (dark blue) and vaccine-derived MAD10–466 (light blue) bound to RH5 (yellow) illustrating similar binding modes. Only the variable domain of the MAD10–466 Fab fragment is shown for simplification. Shared intermolecular contacts made by CDRs H1 and H2 of each mAb are shown in the expanded panel inset, with residues shown as sticks and hydrogen bonds with dashed lines. (D) Comparison of the binding interfaces between MAD8–502 and MAD10–466 to RH5 demonstrating shared use of CDRs H1 and H2 to interact with RH5 but variable use of CDRs H3, L1, L2, and L3. (E) Overlay of doppelgangers MAD8–151 scFv (naturally acquired, dark red) and MAD10–255 (vaccination-derived, red) bound to RH5 (yellow). (F) View of the almost identical binding interface between MAD8–151 and MAD10–255 with RH5. Residues taking part in the binding interface are labeled and shown as sticks, while hydrogen bonds are shown as dashed lines. In the MAD8–151 panel, RH5 residues are numbered as in wild-type RH5 to allow for comparison with MAD10–255, while the deposited structure uses numbering of the RH5ΔNL construct (Table S2). See also Figure S5 and Tables S1 and S2.

11 out of 13 VH genes used by infection-derived mAbs were also used by their vaccination-derived counterparts (Figure 5). Moreover, the two most potent mAbs from natural infection, MAD8–151 and MAD8–502, had similar characteristics to specific vaccine-derived mAbs. MAD8–502 used the same VH gene (IGHV1–69), targeted the same epitope (bin I), and had similar potency to vaccine-derived MAD10–466 (Figure S5B). Infection-acquired MAD8–151 and vaccination-derived MAD10–255 were even more similar, as they shared all five V(D)J genes, had identical lengths for all six complementarity-determining regions (CDRs), targeted the same region of RH5 (bin II), and had very similar potency (Figure S5B). We therefore determined crystal structures of all four of these antibodies, which allowed us to not only compare them with previously characterized antibodies targeting the same epitopes but also to compare them with one another (Figures 6 and S5; Table S1).

To enable crystallization, we prepared scFvs for the naturally acquired antibodies MAD8–502 and MAD8–151 and crystallized complexes of each scFv bound to RH5 (Figures 6A and 6B). As expected from epitope binning, MAD8-502 bound to a site on the top half of RH5, with an epitope consisting of helices 4 and 6 and the loops joining helices 3–4 and 6–7 (Figure 6A). This site overlaps with the binding site for R5.004, with MAD8-502 binding to RH5 with a very similar angle of approach (Figure S5C) and sharing a large number of contact residues (Table S2).^26^ In contrast, MAD8–151 bound to an epitope on the opposite face of the top of RH5, formed from the anti-parallel helices 2 and 3 (Figure 6B). This overlaps with the epitope of R5.016 (Table S2).^26^ However, MAD8–151 binds to RH5 with an angle more like that adopted by the mouse mAb 9AD4^14^ than R5.016 (Figure S5D) and makes more contacts with RH5 helix 3 than R5.016, which has a preference for helix 2.

To enable a comparison with MAD8–502 and MAD8–151, we next determined the structures of vaccine-derived antibodies MAD10–466 and MAD10-255 bound to RH5 using Fab or scFv fragments, respectively, to enable crystallization (Figures S5E and S5F). Aligning the structures of these vaccine-derived antibodies to their naturally acquired counterparts reveals that each pair bind to RH5 with very similar poses (Figures 6C and 6E). In the case of the MAD8–502 and MAD10–466 pair, binding similarity is predominantly within their heavy chains, where interactions made by CDRs H1 and H2 to RH5 are conserved (Figure 6C; Table S2). These CDRs are encoded by the IGHV1-69 gene, which the mAbs share, and utilize a conserved hydrophobic patch in CDR H2 (residues I52, I54, and F/L55) to bind RH5. Although CDRs H3 in both antibodies make several hydrophobic and hydrogen bond interactions with RH5, and share an interaction with R357 of RH5, these occur due to different residues, reflecting differences in gene usage and amino acid sequences despite their CDRs being similar lengths. Indeed, MAD10–466 binds more intimately with RH5 than MAD8–502, with the light chain variable domain rotated slightly toward RH5 (Figure 6C), enabling CDRs L1 to L3 to make contacts (Figure 6D). Meanwhile, only CDR L1 from the light chain of MAD8–502 interacts with RH5 (Figure 6D; Table S2).

The naturally acquired MAD8–151 and vaccine-derived MAD10–255 (bin II), which both target the most potent epitope of RH5,^26^ are remarkably similar, sharing all five V(D)J genes (Figure S5B). An overlay of RH5 bound to MAD8–151 and MAD10–255 shows that these two antibodies bind with an almost indistinguishable binding mode, overlapping with a backbone root-mean-square deviation (RMSD) of 0.57 Å (Figure 6E). Moreover, contact residues between each antibody and RH5 are almost entirely conserved, using the same residues from CDRs H3 and L2-3 (Table S2). Both antibodies make identical hydrogen-bond-mediated binding interactions with D331 of RH5, and several nearly identical interactions with residues F209, K212, N334, N338, and E341 of RH5 (Figure 6F; Table S2). We therefore dubbed MAD8–151 and MAD10–255 “doppelgangers” because they have similar structural and functional properties, but distinct geographical and biological origins. These findings indicate that, at least in these two cases, natural malaria infection is capable of eliciting B cells with similar characteristics to those induced by vaccination with recombinant RH5, despite differences in the mode of antigen exposure and donor origin.

## Discussion

The RH5 vaccine field is at an exciting point in time, with the first clinical trial to test vaccine efficacy in a malaria-endemic region currently ongoing in Burkina Faso (NCT05790889). The vaccination of individuals who have had prior malaria exposure, and could likely have ongoing malaria exposure during or after vaccination, raises questions regarding how the naturally acquired antibody response to RH5 would interact with responses to vaccination. As seen with other pathogens such as the influenza virus and SARS-CoV-2, so-called “hybrid immunity” could lead to synergistic levels of protection, but immune imprinting with obsolete or non-neutralizing regions of surface proteins could instead amplify non-protective antibody responses.^37^^,^^38^^,^^39^ A key prerequisite for evaluating hybrid immunity is first understanding the antibody responses to infection and vaccination separately. Previous reports have suggested that anti-RH5 polyclonal antibody titers in malaria-exposed individuals, including those in this specific cohort, are low^16^^,^^20^^,^^21^^,^^22^ but associate with protection against malaria.^21^^,^^23^^,^^24^^,^^25^ Here, we report the isolation and characterization of RH5-specific mAbs from malaria-exposed individuals, providing a foundation to further explore this research field as clinical trials progress in endemic regions. This study makes three key findings that are relevant to the field.

First, natural infection induces low frequencies of RH5-reactive memory B cells and generates short-lived antibody responses. This observation suggests that exposure to RH5 during *P. falciparum* infection does not often induce productive germinal center responses, although extensive somatic mutations in the infection-derived mAbs that were identified suggest that germinal center maturation can occur. This finding raises questions with respect to whether natural infection would substantially boost vaccine-induced antibody responses. However, the natural boosting effect would likely be more pronounced after vaccination because there would be a larger baseline pool of RH5-reactive B cells triggered by vaccination, as recently observed in Tanzanian infants immunized with a modified vaccinia virus Ankara (MVA) RH5 vaccine.^12^

Second, we identified highly potent mAbs from natural infection that targeted the same RH5 epitopes and used variable genes similar to the best vaccine-induced mAbs identified in this study. Specifically, the doppelgangers MAD8–151 (infection) and MAD10–255 (vaccination) shared IGHV4–59/IGKV1–33, while infection-derived MAD8–502 and vaccine-derived MAD10–466 shared IGHV1–69. These variable genes are distinct from those used by potent circumsporozoite protein (CSP)-specific mAbs that are being developed for clinical use, such as L9 (IGHV3–33/IGKV1–5)^40^ and MAM01 (IGHV3–49/IGLV1–40).^41^ The similarity between infection- and vaccination-derived RH5-specific mAbs could not have been assumed *a priori*, as RH5 is presented differently to the immune system during vaccination and infection. The current RH5 vaccine formulations include the protein as part of a viral-vectored platform,^12^ monomeric protein in adjuvant (ClinicalTrials.gov NCT05790889), or a component of a virus-like particle.^42^ In contrast, *P. falciparum*-expressed RH5 is complexed with at least 4 other malaria proteins, with the full complex only transiently revealed by the merozoite at a critical juncture of invasion followed by rapid engagement of the basigin receptor.^10^ The ability of natural infection to elicit potent antibodies similar to those from vaccination is therefore encouraging for natural boosting of vaccine-induced neutralizing antibody responses, particularly given the data suggesting that potent mAbs can function against *P. falciparum in vivo*.

Third, antibodies from natural infection were more commonly found to target non-neutralizing bottom regions of RH5 distal from the basigin-binding site (bins IV–VI). This observation aligns with previous data suggesting that CyRPA and RIPR detach from RH5 through disassembly of the CyRPA-RH5 interaction upon binding of the RH5 complex to red blood cells,^9^ potentially leaving RH5 attached to basigin with only the bottom of this protein exposed to circulating B cells. The current RH5 vaccines that are being tested in malaria-endemic regions include both top (I–III) and bottom regions of the protein (IV–VI), and a higher frequency of B cells targeting the bottom of RH5 in malaria-exposed individuals could favor the expansion of these non-neutralizing B cells after vaccination. mAbs targeting non-neutralizing sites were unable to trigger Fc effector activity against merozoites in complement and phagocytosis assays, which is consistent with the short duration that RH5 is revealed to the immune system, leaving little time for Fc effectors to engage. Thus, it is unlikely that Fc effector function can overcome the lack of direct neutralization of these mAbs. A potential caveat to the lack of activity of bottom binders applies to a class of mAbs that target bin IV, which do not have neutralizing activity on their own but were previously shown to potentiate the neutralizing activity of specific mAbs targeting the top of RH5.^26^ However, we show here that this comes at the cost of inhibiting the binding of specific neutralizing bin III mAbs to RH5. Considering these factors, we propose that work on a next-generation RH5 vaccine includes a head-to-head comparison between two constructs: one with only top regions of the protein (bins I–III) and one that also includes bin IV. These constructs could be designed either by protein engineering to allow proper folding of the top half of RH5 or by masking non-neutralizing (IV–VI or V–VI) epitopes.

More generally, the analysis of a large panel of mAbs derived from RH5 vaccination and natural infection has provided a higher resolution map of the RH5 surface and allowed us to identify new epitopes that are targets of rarer mAbs. This expanded mAb panel provides a valuable resource for subsequent studies on RH5, including the localization of new mAbs that are discovered in the future. This refinement of the RH5 landscape includes the first structural identification of bin III in complex with a neutralizing mAb. The activation of clonally dispersed B cells targeting different neutralizing epitopes bodes well for development of an RH5 vaccine that is effective in individuals with different genetic backgrounds, as it suggests that the vaccine does not need to selectively trigger rare clones with specific features. Therefore, more complex germline-targeting vaccination approaches such as those done in HIV may not be necessary to develop a successful RH5 vaccine.

### Limitations of the study

A limitation of this study is the relatively few RH5-specific mAbs isolated from malaria-exposed individuals. Despite screening a large number of B cells (∼14 million) from 30 donors that were selected out of a large cohort (*n* = 758) based on high circulating levels of polyclonal RH5-specific antibodies, we were only able to isolate 22 distinct RH5-specific mAbs. The main constraint here was the low frequency of detectable RH5-reactive B cells induced by natural infection. It would be ideal to validate the ratio of RH5-reactive B cells from natural infection targeting the top and bottom of the protein with a larger number of mAbs, but we anticipate that this will require a substantial, dedicated effort to screen extremely large numbers of samples from malaria-exposed donors.

## STAR★Methods

### Key resources table

**Table:** 

REAGENT or RESOURCE	SOURCE	IDENTIFIER
**Antibodies**		

CD14-BV510	Biolegend	Cat. # 301842; RRID: AB_2561946
CD3-BV510	Biolegend	Cat. # 317332; RRID: AB_2561943
CD56-BV510	Biolegend	Cat. # 318340; RRID: AB_2561944
CD19-ECD	Beckman Coulter	Cat. # IM2708U; RRID: AB_130854
IgA-Alexa Fluor 647	Jackson Immunoresearch	Cat. # 109-606-011; RRID: AB_2337895
IgD-PE-Cy7	BD	Cat. # 561314; RRID: AB_10642457
IgM-PerCP-Cy5.5	BD	Cat. # 561285; RRID: AB_10611998
CD27-Alexa Fluor 488	Biolegend	Cat. # 393204; RRID: AB_2750089
CD38-APC-Cy7	Biolegend	Cat. # 303534; RRID: AB_2561605
Anti-human IgG Fc	Jackson Immunoresearch	Cat. # 109-005-098; RRID: AB_2337541
Goat anti-human IgG-Alexa Fluor 647	Jackson Immunoresearch	Cat. # 109-606-170; RRID: AB_2337902
Goat anti-human IgA-Cy3	Jackson Immunoresearch	Cat. # 109-166-011; RRID: AB_2337733
Anti-human CH1 VHH	Thermo Fisher Scientific	Cat. # 7103202100
Anti-human kappa fragment VHH	Thermo Fisher Scientific	Cat. # 7103272100
Anti-human lambda fragment VHH	Thermo Fisher Scientific	Cat. # 7103082100
Anti-His Alexa Fluor 647	Invitrogen	Cat. # MA1-135-A647; RRID: AB_2610637
42D6	Patel et al.^62^	N/A

**Biological samples**		

Cell/Plasma samples from Malian cohort	Tran et al.^27^	N/A
Cell/Serum samples from RH5 vaccine recipients	Minassian et al.^19^	N/A

**Chemicals, peptides and recombinant proteins**		

RH5	Wright et al.^14^ and Crosnier et al.^45^	N/A
CyRPA	Ragotte et al.^35^	N/A
MSP1	Crosnier et al.^45^	N/A
IL21	Gibco	Cat. # PHC0211
R848	Invivogen	Cat. # tlrl-r848
R5.008	Alanine et al.^26^	N/A
Plasma Cell Survival Medium	Berkeley Lights	Cat. # 520-08022
Mycozap	Lonza	Cat. # VZA-2021
Memory B cell Activation Medium	Berkeley Lights	Cat. # 750-08121
Penicillin/Streptomycin	Invitrogen	Cat. # 15140122
Cd4	Crosnier et al.^45^	N/A
OptoSelect 11k chip	Berkeley Lights	Cat. # 500-12012
2.5 μm streptavidin beads, Yellow, Odd # peaks	Spherotech	Cat. # SVFA-2552-6K
2.5 μm streptavidin beads, Yellow, Even # peaks	Spherotech	Cat. # SVFB-2552-6K
2.5 μm streptavidin beads, Pink, Odd # peaks	Spherotech	Cat. # SVFA-2558-6K
2.5 μm streptavidin beads, Pink, Even # peaks	Spherotech	Cat. # SVFB-2558-6K
7 μm streptavidin beads	Spherotech	Cat. # SVP-60-5
CountBright™ Absolute Counting Beads	Thermo Fisher Scientific	Cat. # C36950
LIVE/DEAD Fixable Aqua	Thermo Fisher Scientific	Cat. # L34966
10× HBSTE	Carterra	Cat. # 3630
0.1 M MES pH 5.5	Carterra	Cat. # 3625
Sodium Acetate pH 4.5	Carterra	Cat. # 3802
10% Tween	Carterra	Cat. # 3631
10 mM Glycine pH 2	Carterra	Cat. # 3640
1 M Ethanolamine pH 8.5	Carterra	Cat. # 3626
EDC	Thermo Fisher Scientific	Cat. # PG82079
NHS	Thermo Fisher Scientific	Cat. # 24510
SYBR Green I	Thermo Fisher Scientific	Cat. # S7585
Horseradish peroxidase	Invitrogen	Cat. # A18817
POD substrate	Roche	Cat. # 11582950001
Dynabeads™ mRNA DIRECT™ Purification Kit	Thermo Fisher Scientific	Cat. # 61012
SuperScript™ IV Reverse Transcriptase	Thermo Fisher Scientific	Cat. # 18090010
Human RBC	BloodWorks Northwest, Seattle, WA, USA	N/A
PEIMax	Polysciences	Cat. # 24765-1
FreeStyle™ F17 Expression Medium	Thermo Fisher Scientific	Cat. # A1383501
MEM Non-Essential Amino Acids Solution (100X)	Gibco	Cat. # 11140035
EX-CELL 420 media	Sigma Aldrich	Cat. # 14420C
RPMI-1640	Thermo Fisher Scientific	Cat. # 11875119
DMEM	Thermo Fisher Scientific	Cat. # 11965118
L-glutamine	Gibco	Cat. # 25030081

**Critical commercial assays**		

Pierce Fab Preparation kit	Thermo Fisher Scientific	Cat. # 44985
Expi293F expression system	Thermo Fisher Scientific	Cat. # A14635
ExpiFectamine 293 Transfection Kit	Thermo Fisher Scientific	Cat. # A14524

**Deposited data**		

RH5 mAb sequences (10 most potent)	NCBI GenBank	GenBank: PP840862-PP840881
R5.008 structure	Protein Data Bank (PDB)	PDB: 8PWX
MAD8-151 structure	Protein Data Bank (PDB)	PDB: 8PWW
MAD8-502 structure	Protein Data Bank (PDB)	PDB: 8PWV
MAD10-255 structure	Protein Data Bank (PDB)	PDB: 8PWU
MAD10-466 structure	Protein Data Bank (PDB)	PDB: 8Q5D

**Experimental models: cell lines**		

Irradiated 3T3-CD40L cells	Huang et al.^49^ and Moir et al.^50^	N/A
Expi293 cells	Gibco	Cat. # A14527; RRID: CVCL_D615
FreeStyle™ 293-F cells	Thermo Fisher Scientific	R79007
THP-1 cells	American Type Culture Collection	TIB-202
*Drosophila* S2 cells	ExpreS^2^ion Biotechnologies	N/A

**Experimental models: organisms/strains**		

FRG huHep mice with a NOD background	Yecuris, Inc. (Beaverton, OR, USA)	N/A
*P. falciparum* (strain NF54)	Johns Hopkins Malaria Research Institute Insectary Core	N/A
Mosquitoes	Johns Hopkins Malaria Research Institute Insectary Core	N/A

**Oligonucleotides**		

PCR primers for amplification of antibody heavy, kappa, and lambda genes	Wang et al.^40^	GenBank: MT811859 – MT811914

**Software and algorithms**		

Geneious Prime	https://www.geneious.com	Version 2021.0.3
FlowJo	BD	Version 10.8.1
Cell Analysis Suite	Berkeley Lights	Version 2.4.9.18
GraphPad Prism	Graphpad	Version 9.3.1
iReceptor database	https://gateway.ireceptor.org/home	N/A
Epitope Software	Carterra	N/A
Kinetics Software	Carterra	N/A
International Immunogenetics Information System database (IMGT)	https://www.imgt.org/	N/A
Immcantation Change-O tool	https://changeo.readthedocs.io/en/stable/index.html	N/A

**Other**		

HiTrap Protein A columns	Cytiva/GE Healthcare Life Sciences	Cat. # 17040303
HisTrap HP columns	Cytiva/GE Healthcare Life Sciences	Cat. # 17524802
CaptureSelect CH1-XL Affinity Matrix	Thermo Fisher Scientific	Cat. # 194346205L
ScisGo™	Scisco Genetics	Cat. # HLA-24S-v6
MiSeq Kit v2	Illumina	Cat. # MS-102-2003
iQue Screener Plus	Intellicyt	N/A
Beacon analyzer	Berkeley Lights	N/A
LSA	Carterra	N/A
HC30M chip	Carterra	Cat. # 4279
CMDP chip	Carterra	Cat. # 4282
SAHC30M chip	Carterra	Cat. # 4294
Enspire multi-mode plate reader	PerkinElmer	N/A

### Resource availability

#### Lead contact

Further information and requests for resources and reagents should be directed to and will be fulfilled by the lead contact, Joshua Tan (tanj4@nih.gov).

#### Materials availability

All unique reagents generated in this study are available from the lead contact with a completed Materials Transfer Agreement.

#### Data and code availability

• The heavy and light chain gene sequences of the 10 most potent anti-RH5 human monoclonal antibodies that are the focus of this study have been deposited in GenBank (accession numbers GenBank: PP840862-PP840881) and are publicly available as of the date of publication. Crystal structures of RH5 bound to R5.008, MAD8-151, MAD8-502, MAD10-255 and MAD10-466 are deposited in the Protein Data Bank under accession codes PDB: 8PWX, 8PWW, 8PWV, 8PWU and 8Q5D and are publicly available as of the date of publication. Accession numbers are also listed in the key resources table.
• This paper does not report original code.
• Any additional information required to reanalyze the data reported in this paper is available from the lead contact upon request.

### Experimental model and study participant details

#### Human studies

Clinical specimens were sourced from a longitudinal study of 758 malaria-exposed subjects aged 1 month to 41 years (49.9% female) in the rural village of Kalifabougou, Mali^21^^,^^43^ or the VAC063 study (a multi-center, non-randomized, open-label, dose escalation Phase I/IIa clinical trial evaluating the safety, immunogenicity, and efficacy of RH5.1 formulated with the adjuvant AS01_B_ in 88 healthy, malaria-naïve subjects 18-45 years of age, 62.5% female, in the United Kingdom; ClinicalTrials.gov: NCT02927145).^19^ For the Malian subjects, PBMCs and plasma were isolated from venous blood at the following timepoints: before and after each malaria season, when a malaria infection was diagnosed via referral or biweekly or monthly scheduled visits (acute), and 7-10 days after treatment of the confirmed malaria infection (convalescent). PBMCs from all timepoints were used to isolate mAbs. For the VAC063 subjects, mAbs were isolated from PBMCs collected 12 weeks after the final immunization in Groups 1 and 4 (which respectively received three monthly vaccinations of 2 and 50 μg of RH5.1/AS01_B_). The Kalifabougou cohort study was approved by the Ethics Committee of the Faculty of Medicine, Pharmacy and Dentistry at the University of Sciences, Technique and Technology of Bamako, and the Institutional Review Board of the National Institute of Allergy and Infectious Diseases, National Institutes of Health (NIH IRB protocol number: 11IN126; https://clinicaltrials.gov/; trial number NCT01322581). Written informed consent was obtained from participants or parents or guardians of participating children before inclusion in the study. The RH5 vaccination study was approved by the UK NHS Research Ethics Service (Oxfordshire Research Ethics Committee A, Ref 16/SC/0345), as well as the UK Medicines and Healthcare products Regulatory Agency (Ref 21584/0362/001-0011). Written informed consent was obtained from participants before inclusion in the study.

#### Animal studies

Mouse experiments were performed at Oregon Health and Sciences University (OHSU), which has an approved Assurance (#A3304-01) with the Office for Laboratory Animal Welfare (OLAW), NIH, USA. The experiments were performed under protocol IP00002077, which was approved by the OHSU Institutional Animal Care and Use Committee (IACUC). FRG huHep on NOD mice (female, age 7-8 months) were purchased from Yecuris, Inc. (Beaverton, OR, USA) and confirmed to be successfully humanized by analysis of human albumin levels in serum.

#### Cell lines

HEK293E were obtained from the National Research Council Canada^44^ and were grown in Freestyle media (Thermo Fisher Scientific) at 37°C in a 5% CO2 atmosphere and shaken at 125 rpm. HEK293F lines were cultured in Expi293 expression media (Thermo Fisher Scientific) at 37^o^C, 8% CO_2_, shaking at 125 RPM. *Drosophila* S2 lines were cultured at 25°C in 10% FBS-supplemented EX-CELL 420 media with 100 U/mL penicillin and 0.1 mg/mL streptomycin. 3T3 cells transfected for CD40L expression were cultured in Glutamax-supplemented DMEM (Thermo Fisher Scientific) with 10% FBS and 100 U/mL penicillin and 0.1 mg/mL streptomycin at 37°C and 5% CO_2_ before irradiation at 5000 rads for 5 minutes and 30 seconds. THP-1 cells were cultured in Glutamax-supplemented RPMI-1640 (Thermo Fisher Scientific) with 10% FBS, 0.25% glucose, 1% pyruvate and 50 μM β-mercaptoethanol streptomycin at 37°C and 5% CO_2_.

### Method details

#### Production of recombinant malaria antigens

*P. falciparum* RH5 and MSP1 used for antibody isolation and kinetics assessments were expressed as soluble recombinant proteins by transient transfection of suspension-grown HEK293E cells as described.^45^ In brief, HEK293E were obtained from the National Research Council Canada^44^ and were grown in Freestyle media (Thermo Fisher Scientific) at 37°C, 5% CO2 and shaken at 125 rpm. Expression plasmids encoding the entire ectodomains of each protein were synthesized using codon optimization for human cells and all potential N-linked glycosylation sites removed by mutating appropriate serines or threonines in the context of glycosylation sequons to alanine. Each protein contained a C-terminal rat Cd4 domains 3+4 tag followed by a site for BirA enzymatic biotinylation and a 6-histidine tag for purification. A protein comprising just the rat Cd4, bio, and 6-his tags was used as a control. Proteins were enzymatically monobiotinylated during expression by co-transfection with 1:10 ratio of a plasmid encoding a secreted BirA enzyme (Addgene plasmid #64395).^46^ Purification of the proteins was performed using Ni-NTA resin (GE Healthcare) according to manufacturer’s instructions. Full-length RH5.1 for epitope-binning experiments (residues E26-Q526) and RH5ΔNL used for crystallography (a construct of RH5 encompassing residues K140-K247 and N297-Q526, thereby lacking its flexible N-terminus and internal loop, with substitutions T216A and T299A (to remove potential glycosylation sites) and C203Y (of the 7G8 *Plasmodium falciparum* strain), and a C-terminal His-tag were expressed and secreted from a stable *Drosophila* S2 cell line (ExpreS^2^ion Biotechnologies) in EX-CELL® 420 Serum Free Medium (Sigma Aldrich).^14^ After 3-4 days, the culture supernatant was harvested and adjusted to pH 8 with Tris, spun at 9,000 × g for 15 mins and 0.45 μm filtered, then incubated with Ni Sepharose™ excel resin (Cytiva) for 2 hours. Beads were washed with 5 column volumes of TBS (20 mM Tris pH 8.0, 150 mM NaCl), and 20 column volumes of wash buffer (20 mM Tris pH 8, 500 mM NaCl, 20 mM imidazole), then bound proteins eluted with elution buffer (20 mM Tris pH 8, 150 mM NaCl, 500 mM imidazole). Eluted proteins were diluted 1:1 in ConA binding buffer (20 mM Tris pH 7.5, 500 mM NaCl, 1 mM MnCl_2_, 1 mM CaCl_2_), then incubated with ConA Sepharose 4B resin (Cytiva) overnight at 4°C, after which the unbound fraction containing recombinant protein was recovered. Recombinant protein was further purified by gel filtration using an Superdex 200 Increase 10/300 column into SEC buffer (20 mM HEPES pH 7.5, 150 mM NaCl, 5% glycerol). CyRPA used in RH5 epitope binning experiments was expressed in Expi293F cells as previously described.^35^ Briefly, Expi293F cells were maintained in Expi293 expression medium (Thermo Fisher Scientific) at 37°C, 8% CO2, on an orbital shaker set at 125 RPM. Transfection with CyRPA using ExpiFectamine was maintained for 4 d following manufacturer’s instructions (Thermo Fisher Scientific). CyRPA was purified by C-tag affinity chromatography and subsequently SEC-purified in TBS pH 7.4.

#### Antibody binding to antigen-coated beads

Streptavidin beads (Spherotech) were incubated with biotinylated RH5 (50 μg/mL) and MSP1 (10 μg/mL) for 30 minutes at room temperature, washed with PBS + 0.5% BSA, blocked with CD4 (10 μg/ml; negative control antigen) for 30 minutes at room temperature, washed, mixed, aliquoted, and frozen at -80^o^C. To measure plasma binding, 1:20 diluted Malian plasma was incubated with thawed antigen-coated beads for 30 minutes at room temperature, washed, and stained with 2.5 μg/mL goat anti-human IgG Alexa Fluor-647 (AF647) secondary antibody (Jackson Immunoresearch). To measure mAb binding, eight mAb dilutions (25, 3.57, 0.51 μg/mL, etc.; 7-fold serial dilutions) were incubated with antigen-coated beads for 30 minutes at room temperature, washed, and incubated with IgG-AF647. After a final wash, plasma- or mAb-bound beads were read with the iQue Screener Plus (Intellicyt) high-throughput flow cytometer and data were analyzed with FlowJo (Tree Star). Malian plasma data were normalized by dividing RH5 or MSP1 median fluorescence intensity (MFI) by CD4 MFI. For mAb titrations, AUC values were calculated using GraphPad Prism and normalized based on interexperimental variance of positive control RH5-specific mAb R5.016^26^ with the first experiment’s R5.016 AUC values for RH5 binding being used as the anchors by which all subsequent R5.016 values were divided; anti-HIV-1 mAb VRC01^47^ was included as a negative control.

#### Isolation of RH5-specific B cells

RH5-specific memory B cells (MBCs) and plasmablasts (PBs) were identified using the Beacon Optofluidic System as previously described.^48^ Briefly, cryopreserved PBMCs were thawed and stained for 20 minutes at 4°C in PBS with LIVE/DEAD Fixable Aqua (Thermo Fisher Scientific) prior to staining for 20 minutes at 4°C in PBS + 1% FBS with the following panel: CD3-BV510, CD14-BV510, CD27-AF488, CD38-APC/Cy7, CD56-BV510 (BioLegend), CD19-ECD (Beckman Coulter), IgA-AF647 (Jackson Immunoresearch), IgM-PerCP-Cy5.5, and IgD-PECy7 (BD Biosciences). Sorted single CD3^-^CD14^-^CD56^-^CD19^+^IgD^-^IgM^-^IgA^-^ MBCs were cultured via two methods: 1) 2,500 MBCs/well cultured in a proprietary cytokine cocktail (Berkeley Lights) for 6 days in 96-well U-bottom plates or, 2) 100-250 MBCs/well and 10,000 3T3-CD40L feeder cells/well^49^^,^^50^ cultured in I10 media (Iscove’s modified Dulbecco’s Medium, 10% FBS, 1:1000 MycoZap; Thermo Fisher and Lonza) supplemented with 100 ng/mL IL-21 (Gibco) and 0.5 μg/mL R848 (Mabtech) for 10 days in 384-well plates. After stimulation, culture supernatants were screened for binding to antigen-coated beads as described above. MBCs from positive wells were suspended in either Plasma Cell Survival Media (PCSM, Berkeley Lights) or I10 media supplemented with IL-21 and R848, loaded onto an OptoSelect 11k chip, and individually moved into nanoliter-volume pens using opto-electropositioning (OEP) light cages drawn by the Beacon’s Cell Analysis Suite (Berkeley Lights). Channels were then flooded with 7 μm streptavidin beads (Spherotech, SVP-60-5) coated with 50 μg/mL of biotinylated RH5 and suspended in a cocktail of 2.5 μg/mL IgG-AF647. Over a 30-minute time course, B cells producing RH5-specific antibodies were detected by the appearance of fluorescent ‘blooms’. Live singlet CD3^-^CD14^-^CD56^-^CD19^+^CD27^+^CD38^+^ PBs were sorted into PCSM and screened in a similar manner as above in PCSM. Positive B cells were individually exported using OEP light cages into 96-well plates containing lysis buffer and frozen at −80°C.

#### mAb sequence analysis and production

PCR amplification and sequencing of VH and VL chains was done as previously described.^40^^,^^48^ Briefly, RNA from lysed B cells was cleaned up (Dynabeads™ mRNA DIRECT™ Purification Kit, Thermo Fisher Scientific) and reverse transcribed to cDNA (SuperScript™ IV Reverse Transcriptase, Thermo Fisher Scientific). The cDNA was then subjected to specific amplification of the genes encoding the immunoglobulin variable regions heavy and light chains using a cocktail of primers (primer sequences available in GenBank MT811859 – MT811914), followed by sequencing (ACGT) and cloning into expression vectors (GenScript) containing the relevant constant region. Sequence analysis was performed by importing FASTA sequences for heavy and light chains into the International Immunogenetics Information System (IMGT) database, which yielded predicted genetic background, CDR1-3 sequences and antibody isotype.^51^ Clonal analysis of the mAb panel was performed with SHazaM and Spectral Clustering for clOne Partitioning (SCOPer) from the Immcantation analysis framework.^52^^,^^53^ Briefly, the VH sequences were compiled in FASTA format and converted into an .airr file type using the Immcantation Change-O tool (https://changeo.readthedocs.io/en/stable/index.html). Next, the VH sequences were analyzed by SHazaM to determine the threshold value for grouping the sequences into clonal families, using the gamma/Gaussian mixture method (gmm) model. SCOPer was then used to perform hierarchical clustering to determine the clonal relationship between the mAbs, using the provided threshold value from SHazaM. Only MAD8-652 could not be analyzed in this system, but this mAb was unambiguously identified as belonging to a unique clone as it used a different VH gene and CDRH3 length from all other mAbs isolated from its source donor. Antibody VH or VL sequences were cloned into plasmids containing an IgG1 or relevant light chain backbone (GenScript) and transfected into Expi293 cells (Thermo Fisher Scientific). HiTrap Protein A columns (GE Healthcare Life Sciences) were used to purify recombinant IgG. For Fab production, heavy chain plasmids encoding only VH and CH1 (domain 1 of the constant region of the immunoglobulin heavy chain) were synthesized and used to transfect Expi293 cells alongside light chain plasmids. Fab fragments were purified by anti-CH1 resin (Thermo Fisher Scientific). scFv fragments for MAD8-151, MAD8-502 and MAD10-255, consisting of VH and VL domains joined by a flexible 15-residue long glycine-rich linker sequence, were transiently expressed in Expi293 cells and purified by His-tag affinity columns. An scFv construct of R5.008^26^ comprising the variable heavy chain and light chains joined with the 15-residue linker was transiently expressed in a secreted form using FreeStyle™ 293-F cells (Thermo Fisher) in FreeStyle™ F17 Expression Medium supplemented with L-glutamine and 1× MEM non-essential amino acids (Gibco). After 6 days, cultures were purified by His-tag affinity as performed for the other scFv fragments. To isolate monomeric scFv, eluted proteins were further purified by gel filtration on an S200 Increase 10/300 into 20 mM HEPES pH 7.5, 150 mM NaCl at room temperature.

#### HLA typing of donor cDNA

When B cells from multiple donors were pooled for optofluidic screening, HLA-typing was required to match isolated mAbs to donor source. HLA-typing was carried out using a two-stage PCR for locus amplification and sample barcoding as described previously.^29^ Briefly, cell isolates were subjected to sequential Stage 1 and Stage 2 amplification using cDNA-specific amplicons from the ScisGo®-HLA-v6 kit (Scisco Genetics Inc., Seattle WA). Resulting reactions were then purified and subjected to MiSeq using Illumina Version 2 chemistry with 500-cycle, paired-end sequencing (Illumina, San Diego, CA). Data were assembled with Sciscloud® software (Scisco Genetics Inc., Seattle WA); computational tools were adapted specifically for HLA genomic assembly from reactions amplified with the ScisGo®-HLA-v6 kit. For unambiguous typing of corresponding samples, amplified portions of HLA class I and II genes were matched to prior typing data. All software used for HLA typing was included with kit components.

#### Antibody affinity measurements

All antibody affinity experiments were performed with the Carterra LSA. A CMDP chip (Carterra 4282) was primed with 25 mM MES buffer. The chip was conditioned with 50 mM NaOH, 500 mM NaCl and 10 mM Glycine (pH 2.0) (Carterra 3640), and activated with a mixture of 133 mM 1-ethyl-3-(3-dimethylaminopropyl) carbodiimide hydrochloride and 33 mM N-hydroxysuccinimide (Thermo Fisher Scientific). The activated chip was lawned with goat anti-human IgG Fc (50 μg/mL) (Jackson ImmunoResearch) in 10 mM sodium acetate (pH 4.5) (Carterra 3802) with 0.05% Tween (Carterra 3631) before blocking with 1 M ethanolamine (pH 8.5) (Carterra 3626) and washing with 10 mM Glycine (pH 2.0). Next, mAbs were diluted to 100 ng/mL in HEPES-buffered saline Tween-EDTA (HBSTE) with 0.5mg/mL BSA. This array was printed onto the chip by capture. RH5 was then injected onto bound mAbs in eight increasing concentrations, up to a top concentration of 56 nM, following a 10-minute association and 30-minute dissociation protocol. The SPR results were exported to Kinetics Software (Carterra) and analyzed as nonregenerative kinetics data to calculate association rate constant (K_a_), dissociation rate constant (K_d_), and equilibrium dissociation constant (K_D_) values. To confirm that RH5 kinetics could be measured by using either Fab or IgG, we selected one mAb from each of bins I-VI and expressed these six mAbs as Fab fragments. These were then captured on a SAHC30M chip (Carterra 4294) pre-conditioned with 25 mM NaOH, 1 M NaCl and 10 mM Glycine (pH 2.0) and pre-lawned with 10 μg/mL anti-human CH1 (Thermo Fisher Scientific 7103202100), 10 μg/mL anti-human kappa-fragment (Thermo Fisher Scientific 7103272100) and 10 μg/mL anti-human lambda-fragment (Thermo Fisher Scientific 7103082100). Fab fragments were printed at double the molarity of IgG molecules to account for the fact that IgG molecules possess two epitope-binding sites as opposed to one for Fab fragments. The RH5 kinetics protocol was the same as described above, except that association was reduced to 5 minutes and dissociation to 10 minutes. Data were processed with Kinetics software.

#### mAb epitope determination

##### Epitope binning

Epitope binning experiments were performed with the Carterra LSA. A HC30M chip (Carterra 4279) was primed with 25 mM MES buffer, conditioned with 50 mM NaOH, 500 mM NaCl and 10 mM Glycine (pH 2.0) (Carterra 3640), and activated with a mixture of 133 mM 1-ethyl-3-(3-dimethylaminopropyl) carbodiimide hydrochloride and 33 mM N-hydroxysuccinimide (Thermo Fisher Scientific). The activated chip was coupled directly to mAbs diluted to 10 μg/mL in 10 mM sodium acetate (pH 4.5) (Carterra 3802) with 0.05% Tween (Carterra 3631), before blocking with 1 M ethanolamine (pH 8.5) (Carterra 3626) and washing with 10 mM Glycine (pH 2.0). The coupled antibody array was then subjected to sequential injections of 50 nM RH5 and 10 μg/ml sandwiching antibody, both diluted to final concentration in HBSTE with 0.5 mg/mL BSA. The protocol followed involved 5 minutes for antigen injection, 5 minutes for monoclonal antibody injection and 1 minute for dissociation, before glycine-mediated chip regeneration, allowing for the injection of the next antibody in the array. The SPR results were exported to Epitope Software (Carterra) to identify competing or sandwiching pairs of monoclonal antibodies.

##### Epitope localization assay

Streptavidin beads (Spherotech) were incubated with biotinylated RH5 (50 μg/mL) for 30 minutes at room temperature, washed with PBS + 0.5% BSA, blocked with CD4 (10 μg/ml; negative control antigen) for 30 minutes at room temperature, washed, and subsequently incubated with high-affinity anti-RH5 mAbs expressed in the IgA1 isotype. Several bead populations with differing intrinsic fluorescent intensities were used to set up this multiplex assay. One set of beads was exclusively incubated with top-binding anti-RH5 mAbs, another with only bottom-binding anti-RH5 mAbs, and yet another with both top and bottom-binding anti-RH5 mAbs. A fourth set of beads was left unblocked. After 30 minutes incubation, bead batches were washed and mixed. All anti-RH5 mAbs, in IgG format, were incubated with these bead mixes for 30 minutes at room temperature. A number of mAb concentrations were assessed to account for differences in binding strength between mAbs. Beads were subsequently washed and stained with 2.5 μg/mL goat anti-human IgG-AF647 secondary antibody (Jackson Immunoresearch). After a final wash, mAb-bound beads were read with the iQue Screener Plus (Intellicyt) high-throughput flow cytometer and data were analyzed with FlowJo (Tree Star). Epitope localization score was calculated as the binding MFI of mAb to bottom-blocked beads divided by binding MFI to top-blocked beads.

#### Crystallization and structure determination

To prepare complexes for crystallization, RH5ΔNL was treated with 1 μg/mL Endoproteinase Glu-C (Sigma) overnight at room temperature, then combined with scFv fragments of R5.008, MAD8-151, MAD8-502 or MAD10-255, or the Fab fragment of MAD10-466, in each case with RH5ΔNL in slight molar excess. Complexes were isolated by gel filtration using an S200 Increase 10/300 column running in SEC buffer (20 mM HEPES pH 7.5, 150 mM NaCl, 5% glycerol), then concentrated using 10K Amicon® Ultra centrifugal units. Complexes were crystallized by sitting drop vapour diffusion at 18°C mixing 100 nL each of protein and reservoir solution for all complexes except for R5.008 and MAD10-466, which are detailed below.

Crystals of RH5ΔNL:R5.008 were obtained at 7 mg/mL in Morpheus E11 (0.1 M Buffer System 3 pH 8.5, 0.12 M Ethylene Glycol Mix, 30% v/v Precipitant Mix 3) containing Silver Bullets G6 (0.02 M HEPES pH 6.8, 0.16% w/v Glutaric acid, 0.16% Mellitic acid, 0.16% Oxalic acid anhydrous, 0.16% Pimelic acid, 0.16% Sebabic acid, 0.16% trans-Cinnamic acid), mixing 100 nL protein, 100 nL reservoir solution and 50 nL Silver Bullets additive. Crystals of RH5ΔNL:MAD10-466 were obtained at 7.5 mg/mL in Morpheus H9 (0.1 M Buffer System 3 pH 8.5, 0.1 M Amino Acids Mix, 30% v/v Precipitant Mix 1) containing Hampton Additive G8 (40% v/v Polypropylene glycol P 400), mixing 100 nL protein, 50 nL reservoir solution, 50 nL additive, and 50 nL crystal seeds prepared using Seed Beads™ (Hampton Research) from crystals obtained in Morpheus H9 alone. Crystals of RH5ΔNL:MAD8-151 were obtained at 10 mg/mL in ProPlex E9 (0.2 M sodium chloride, 0.1 M sodium HEPES pH 7.5, 12% w/v PEG 8000), of RH5ΔNL:MAD8-502 at 11 mg/mL in JCSG+ D12 (0.04 M potassium phosphate monobasic, 16% w/v PEG 8000, 20% w/v glycerol), and of RH5ΔNL:MAD10-255 at 9 mg/mL in PGA H12 (0.1 M ammonium sulphate, 0.3 M sodium formate, 0.1 M Tris pH 7.8, 2% w/v γ-PGA (Na+ form, LM), 3% w/v PEG 20000). For data collection, crystals were cryoprotected where required in reservoir solution supplemented with 25% glycerol, then cryo-cooled in liquid nitrogen. Data were collected at Diamond Light Source on beamline I24 (wavelength 0.9999 Å) for RH5ΔNL:R5.008 and beamline I04 (wavelength 0.95374 Å) for all others.

Diffraction data for RH5ΔNL:R5.008 was auto-processed with xia2-multiplex combining two datasets acquired from the same crystal at different locations. Frames 1501-1800 of dataset 1 and frames 11501-11800 of dataset 2 were excluded due to radiation damage. The combined dataset was reduced, scaled, and merged using AIMLESS in CCP4i2^54^ to 3.20 Å. Data for RH5ΔNL:MAD8-151 was auto-processed using autoPROC to 1.95 Å, and data for RH5ΔNL:MAD8-502, RH5ΔNL:MAD10-255 and RH5ΔNL:MAD10-466 were auto-processed in xia2-dials to 2.07 Å, 3.15 Å and 3.20 Å respectively. Molecular replacement for each dataset except RH5ΔNL:MAD10-466 was performed using PHASER in the CCP4 suite, using RH5 from PDB 6RCU and an scFv scaffold as search models. Molecular replacement of the MAD10-466 dataset was performed using the structure of RH5-bound MAD8-502 determined in this study and the constant domain of a Fab fragment (PDB ID 7PHU), also in PHASER. Models were built and refined in cycles using COOT (0.9.3),^55^ BUSTER (2.10.4)^56^ and PHENIX (1.20.1-4487).^57^ The unit cell for RH5ΔNL:R5.008 contains one copy of the RH5:scFv complex, while RH5ΔNL:MAD8-151 contains two copies, and RH5ΔNL:MAD8-502 contains four copies. RH5ΔNL:MAD10-255 contains five complete copies of the RH5:scFv complex and a further copy of RH5 alone with low occupancy for its partner scFv (which has therefore been omitted). The unit cell for RH5ΔNL:MAD10-466 contains two copies of the RH5:Fab fragment complex. For structural analysis, chains A and B for each RH5:scFv complex were used, or chains A, B and C for the RH5ΔNL:MAD10-466 Fab complex, with other chains in some cases showing weaker density. Binding interfaces were analyzed using PDBe PISA,^58^ and structures were visualized and rendered in ChimeraX.^59^

#### *In vitro* functional assays

##### Growth inhibition activity (GIA) assay

The GIA was performed by the GIA Reference Center, NIH, USA as previously described.^60^ Briefly, all test antibodies were dialyzed against RPMI 1640. The indicated concentration of a test antibody was mixed with a trophozoite-rich 3D7 clone of *P. falciparum* in a 96-well plate (a total of 40 μL/well with 1% hematocrit at ∼0.3% parasitemia in the final well). The plate was incubated in an atmosphere of 5% O_2_, 5% CO_2_, and 90% N_2_ for ∼40 hours at 37°C. After the incubation, relative parasitemia in each well was quantified by parasite-specific lactate dehydrogenase (LDH) activity. The optical density at 650 nm (OD_650_) for each well was read by a VersaMax microplate reader and the % GIA value of each test sample was calculated as:

$$\%\;\text{GIA}=1-\;\left({\text{OD}}_{650}\text{test}\;–\;{\text{OD}}_{650}\text{uRBC}\right)\;/\;\left({\text{OD}}_{650}\text{iRBC}\;–\;{\text{OD}}_{650}\text{uRBC}\right)\times 100$$

where OD_650_test, OD_650_uRBC, and OD_650_iRBC are average OD_650_ values for test antibody, uninfected RBC alone (no test antibody), and infected RBC alone (no test antibody) wells, respectively.

The antigen-reversal GIA was conducted as described elsewhere.^32^ Briefly, a test antibody was mixed with 0.5 mM of RH5 in a 96-well plate (RPMI 1640 was used as the buffer both for test antibody and RH5), then incubated for 45 minutes at room temperature followed by a 15 minute incubation at 37°C. As done in the regular GIA described above, trophozoite-rich *P. falciparum* was added to the plate (a total of 40 μL/well with 1% hematocrit at ∼0.3% parasitemia in a final well), then the LDH assay was performed after a ∼40-hour incubation at 37°C.

To determine the effect of complement on inhibitory activity of the RH5-specific mAbs, a modified GIA was performed. Before initiation of the modified GIA, one set of parasites was cultured using culture medium with 25% non-heat-inactivated human serum, and another set of parasites was cultured using medium with 25% heat-inactivated human serum (regular culture medium contains 10% heat-inactivated human serum), with repeat sorbitol synchronization as for a regular GIA, for a week. After the “adaptation”, the modified GIA was performed with 25% human serum medium (either non-heat-inactivated or heat-inactivated). Other steps were identical to that of the standard GIA.

##### Phagocytosis assay

Merozoites used in phagocytosis assays were freshly harvested from ongoing blood stage parasite cultures as described elsewhere.^61^ THP-1 cells were bought from the American Type Culture Collection and were extracted from ongoing culture and not thawed from frozen stocks. Fresh merozoites were stained with SYBR Green I (Thermo Fisher Scientific S7585) at 1:2000 dilution and quantified using counting beads (Thermo Fisher Scientific C36950). mAbs of interest were diluted for tenfold titration curves from 100 μg/mL to 100 pg/mL. The potent anti-MSP1 antibody 42D6^62^ was included as a positive control and an anti-SARS-CoV-2 antibody was used as a negative control. 0.8 × 10^6^ merozoites and 0.1 × 10^6^ THP-1 cells were seeded per well of a round-bottom 96-well plate with dilutions of antibody. Wells were incubated at 37^o^C with 5% CO_2_ for 40 minutes before centrifugation at 1200 × g to cease phagocytosis and washing in 10% FBS. Phagocytosis was measured as % SYBR Green I-positive THP-1 cells as assessed by FACS. To account for non-specific adherence of merozoites to the exteriors of THP-1 cells, an average of two blank wells (THP-1 cells seeded with merozoites and no antibody) was subtracted from all samples.

#### Murine challenge experiments

Challenge studies were carried out as described previously.^63^ Briefly, FRG huHep on NOD mice were purchased from Yecuris, Inc. (Beaverton, OR, USA) and confirmed to be successfully humanized by analysis of human albumin levels in serum. Mosquitoes for the infection were obtained from the Johns Hopkins Malaria Research Institute Insectary Core. Mice were then infected with *P. falciparum* (strain NF54) by mosquito bite; each mouse was anesthetized under isoflurane for 10 minutes and caged with 5 infectious mosquitoes. Five days post-infection (p.i.), mice were injected intraperitoneally with human RBC (BloodWorks Northwest, Seattle, WA, USA) and intravenously with MAD8-151 or the negative control mAb 1245.^30^ Circulating levels of human RBC were maintained by injection of additional RBC on days 6, 9 and 11 p.i. Blood was collected from mice on days 6, 7, 9, 11 and 13 p.i. Terminal serum was collected on day 13 p.i. by cardiac puncture. Dr. Sean Murphy at the University of Washington quantified *Plasmodium* 18s rRNA by reverse-transcriptase quantitative PCR (RT-qPCR) as previously described.^64^ Briefly, RNA extraction and amplification were performed with the Abbott m2000sp and m2000rt instruments (Abbott Molecular). Blood samples from mice were added to NucliSENS lysis buffer (bioMérieux) and the lysate was prepared for RT-qPCR using the mSample RNA preparation kit following manufacturer’s instructions (Abbott Molecular). 18S rRNA genes were amplified using both pan-*Plasmodium* and *P. falciparum*-specific primers, along with a human housekeeping mRNA target as a control, using the SensiFAST™ Probe Lo-ROX One-Step Kit (Bioline).

### Quantification and statistical analysis

Correlation r and P values for associations between antibody levels and age in the Malian cohort were generated by Spearman correlation. Comparisons between paired acute and convalescent samples were made by Wilcoxon sign-rank test. Comparisons between infection- and vaccination-derived mAbs were done by the Mann-Whitney U test. Spearman correlations for potency of mAbs in bins I and II with sequence and binding parameters was determined in R (*corrplot* package).^65^ r and P values for correlations between RH5 binding (AUC) and growth inhibition percentage were generated by Spearman correlation. Statistical details for each experiment (including exact value of n, choice of statistical test and measurements of center and dispersion of data) can be found in figure legends.

## Supplemental information

Table S1. Crystallographic data collection and refinement statistics, related to Figure 6

Table S2. Table of contacts between R5.008, MAD8–502, MAD10–466, MAD8–151, and MAD10–255 with RH5, related to Figure 6
