# Supplementary material for: Natural malaria infection elicits rare but potent neutralizing antibodies to the blood-stage antigen RH5
Source: Cell. 2024 Sep 5;187(18):4981–4995.e14. doi: 10.1016/j.cell.2024.06.037 (PMC11383431; doi:10.1016/j.cell.2024.06.037)
Supplement: Table S1. Crystallographic data collection and refinement statistics, related to Figure 6 [file mmc1.pdf]

Cell, Volume *187*

Supplemental information

Natural malaria infection elicits rare but potent neutralizing antibodies to the blood-stage antigen RH5

Lawrence T. Wang, Andrew J.R. Cooper, Brendan Farrell, Kazutoyo Miura, Ababacar Diouf, Nicole Müller-Sienerth, Cécile Crosnier, Lauren Purser, Payton J. Kirtley, Maciej Maciuszek, Jordan R. Barrett, Kirsty McHugh, Rodney Ogwang, Courtney Tucker, Shanping Li, Safiatou Doumbo, Didier Doumtabe, Chul-Woo Pyo, Jeff Skinner, Carolyn M. Nielsen, Sarah E. Silk, Kassoum Kayentao, Aissata Ongoiba, Ming Zhao, Doan C. Nguyen, F. Eun-Hyung Lee, Angela M. Minassian, Daniel E. Geraghty, Boubacar Traore, Robert A. Seder, Brandon K. Wilder, Peter D. Crompton, Gavin J. Wright, Carole A. Long, Simon J. Draper, Matthew K. Higgins, and Joshua Tan

Table S1. Crystallographic data collection and refinement statistics, related to Figure 6

	RH5ΔNL:R5.008	RH5ΔNL:MAD8-151	RH5ΔNL:MAD8-502	RH5ΔNL:MAD10-255	RH5ΔNL:MAD10-466
**Data Collection**					
Space group	P 31 2 1	P 1 21 1	P 1 21 1	P1 21 1	P 1 21 1
Cell dimensions					
a, b, c (Å)	114.99, 114.99, 147.49	78.30, 121.38, 79.00	85.97, 158.01, 113.02	115.31, 134.52, 198.67	82.46, 140.41, 83.62
α, β, γ (°)	90, 90, 120	90, 90.18, 90	90, 94.59, 90	90, 93.56, 90	90, 112.74, 90
Wavelength	0.9999 Å	0.95374	0.95374	0.95374	0.95374
Resolution (Å)	57.49 – 3.20 (3.42-3.20)	79.00-1.95 (1.98-1.95)	79.01-2.07 (2.11-2.07)	87.45-3.15 (3.20-3.15)	67.59-3.20 (3.26-3.20)
Total Observations	306591 (55698)	730651 (30772)	1245190 (41161)	739821 (34690)	205662 (10293)
Total Unique	19138 (3408)	106577 (5404)	181986 (8654)	104997 (5215)	28799 (1404)
R_{merge} (%)	49.6 (301.0)	25.2 (397.6)	12.6 (328.2)	19.0 (231.3)	55.1 (291.3)
R_{meas} (%)	51.4 (311.4)	27.3 (437.3)	13.6 (369.7)	20.5 (251.0)	59.4 (313.5)
R_{pim} (%)	13.2 (78.3)	10.4 (180.2)	5.2 (166.4)	7.7 (97.0)	22.1 (115.4)
$CC_{1/2}$	0.986 (0.732)	0.993 (0.337)	0.997 (0.370)	0.996 (0.264)	0.952 (0.345)
$I/\sigma(I)$	5.2 (1.0)	6.0 (0.7)	7.5 (0.2)	7.3 (0.4)	2.3 (0.3)
Completeness (%)	100.00 (99.9)	98.5 (100.0)	99.7 (95.0)	100.0 (99.1)	99.3 (97.0)
Multiplicity	16.00 (16.3)	6.9 (5.7)	6.8 (4.8)	7.0 (6.7)	7.1 (7.3)
Wilson B factor	70.8	27.9	49.4	102.5	74.0
**Refinement**					
Reflections	19053	105428	175452	104799	28644
Rwork / Rfree (%)	24.02/29.35	23.01/25.15	26.87/27.91	24.19/26.36	27.19/31.67
Average B factor					
Protein (all)	90.4	32.6	72.2	136.0	100.89
Protein (Chain A)	82.4	34.4	75.3	115.7	94.53
Protein (Chain B)	96.2	29.5	61.0	106.0	90.31
Protein (Chain C)	-	-	-	-	20.07
Water	74.0	51.6	63.6	n/a	n/a
Ligands	70.7	37.3	n/a	n/a	n/a
Number of residues					
Protein	516	1034	2038	2848	1383
Water	6	533	418	0	0
Ligands	1	7	0	0	0
RMSDs					
Bond lengths (Å)	0.008	0.008	0.008	0.009	0.007
Bond angles (°)	0.97	0.95	0.98	1.03	0.92
Ramachandran plot					
Favored (%)	92.0	97.8	97.9	93.2	92.1
Allowed (%)	8.0	2.0	2.1	6.8	7.9
Outliers (%)	0.0	0.2	0.0	0.0	0.0
