# Supplementary material for: Natural malaria infection elicits rare but potent neutralizing antibodies to the blood-stage antigen RH5
Source: Cell. 2024 Sep 5;187(18):4981–4995.e14. doi: 10.1016/j.cell.2024.06.037 (PMC11383431; doi:10.1016/j.cell.2024.06.037)
Supplement: Table S2. Table of contacts between R5.008, MAD8–502, MAD10–466, MAD8–151, and MAD10–255 with RH5, related to Figure 6 [file mmc2.pdf]

Table S2. Table of contacts between R5.008, MAD8-502, MAD10-466, MAD8-151 and MAD10-255 with RH5, related to Figure 6

		R5.008					
Chain	Residue	Group	Location	Chain	Residue	Group	Interaction
A	D59	Side chain	CDR H2	B	K457	Side chain	Salt bridge
A	Y103	Side chain	CDR H3	B	D440	Side chain	H bond
A	Y104	Side chain	CDR H3	B	N445	Side chain	H bond
		Side chain	CDR H3	B	D461	Side chain	H bond
		Side chain	CDR H3	B	R458	Side chain	Cation-π
A	S106	Side chain	CDR H3	B	R458	Side chain	H bond
A	Q164	Side chain	CDR L1	B	N349	Side chain	H bond
A	Y169	Side chain	CDR L1	B	R448	Side chain	H bond
A	Y229	Backbone	CDR L3	B	R448	Side chain	H bond
		Side chain	CDR L3	B	T449	Side chain	H bond
		Side chain	CDR L3	B	N349	Backbone	H bond
A	T231	Side chain	CDR L3	B	D453	Side chain	H bond

		MAD8-502					
Chain	Residue	Group	Location	Chain	Residue	Group	Interaction
B	T28	Side chain	CDR H1	A	H365	Side chain	H bond
B	S31	Side chain	CDR H1	A	D361	Side chain	H bond
		Backbone	CDR H1			Side chain	H bond
		Backbone	CDR H1	A	W447	Side chain	H bond
B	I52	Side chain	CDR H2				Hydrophobic
B	I54	Side chain	CDR H2				Hydrophobic
B	L55	Side chain	CDR H2				Hydrophobic
B	R57	Side chain	CDR H2	A	W447	Backbone	H bond
B	D101	Side chain	CDR H3	A	Y358	Side chain	H bond
		Side chain	CDR H3	A	R357	Side chain	Salt bridge
B	P103	Side chain	CDR H3				Hydrophobic
B	F104	Side chain	CDR H3				Hydrophobic
B	S164	Backbone	CDR L1	A	N347	Side chain	H bond
B	D165	Side chain	CDR L1	A	N352	Side chain	H bond
				A	Y358	Side chain	Hydrophobic
				A	K443	Side chain	Hydrophobic
				A	W447	Side chain	Hydrophobic
				A	I446	Side chain	Hydrophobic
				A	I442	Side chain	Hydrophobic

		MAD10-466					
Chain	Residue	Group	Location	Chain	Residue	Group	Interaction
B	T28	Side chain	CDR H1	A	H365	Side chain	H bond
B	S31	Side chain	CDR H1	A	D361	Side chain	H bond
	S31	Backbone	CDR H1	A	D361	Side chain	H bond
	S31	Backbone	CDR H1	A	R357	Side chain	H bond
B	S31	Backbone	CDR H1	A	W447	Side chain	H bond
B	I52	Side chain	CDR H2				Hydrophobic
B	I54	Backbone	CDR H2	A	K443	Side chain	H bond
	I54	Side chain	CDR H2				Hydrophobic
B	F55	Side chain	CDR H2				Hydrophobic
B	H99	Backbone	CDR H3	A	R357	Side chain	H bond
B	G100	Backbone	CDR H3	A	R357	Side chain	H bond
B	Y101	Side chain	CDR H3	A	D361	Side chain	H bond
	Y101	Backbone	CDR H3	A	N354	Side chain	H bond
	Y101	Backbone	CDR H3				Hydrophobic
B	S103	Side chain	CDR H3	A	N352	Side chain	H bond
	S103	Side chain	CDR H3	A	N354	Side chain	H bond
C	R30	Side chain	CDR L1	A	F350	Side chain	Cation-π
C	D50	Side chain	CDR L2	A	N352	Side chain	H bond

C	N53	Side chain	CDR L2	A	S197	Side chain	H bond
C	S67	Side chain	FWR 3	A	Y346	Side chain	H bond
C	N93	Side chain	CDR L3	A	W447	Backbone	H bond
				A	I442	Side chain	Hydrophobic
				A	I446	Side chain	Hydrophobic
				A	W447	Side chain	Hydrophobic

MAD8-151							
**Chain**	**Residue**	**Group**	**Location**	**Chain**	**Residue †**	**Group**	**Interaction**
B	S31	Side chain	CDR H1	A	N334 (N285)	Side chain	H bond
		Side chain	CDR H1	A	N334 (N285)	Backbone	H bond
B	Y32	Side chain	CDR H1	A	T337 (T288)	Side chain	H bond
		Side chain	CDR H1	A	E341 (E292)	Side chain	H bond
B	Y33	Side chain	CDR H1	A	D331 (D282)	Side chain	H bond
B	Y52	Side chain	CDR H2	A	K327 (K278)	Side chain	H bond
B	H53	Side chain	CDR H2	A	D331 (D282)	Side chain	Salt bridge
B	R97	Side chain	CDR H3	A	N338 (N289)	Side chain	H bond
B	V99	Backbone	CDR H3	A	N334 (N285)	Side chain	H bond
B	M101	Backbone	CDR H3	A	D331 (D282)	Side chain	H bond
B	I102	Backbone	CDR H3	A	D331 (D282)	Side chain	H bond
		Side chain	CDR H3				Hydrophobic
B	D184	Side chain	CDR L2	A	K212 (K212)	Side chain	Salt bridge
B	Y225	Side chain	CDR L3	A	K212 (K212)	Side chain	H bond
				A	F209 (F209)	Side chain	Hydrophobic
				A	I213 (I213)	Side chain	Hydrophobic
				A	A216 (A216)	Side chain	Hydrophobic
				A	I328 (I279)	Side chain	Hydrophobic

MAD10-255							
**Chain**	**Residue**	**Group**	**Location**	**Chain**	**Residue**	**Group**	**Interaction**
B	T31	Side chain	CDR H1	A	N334	Side chain	H bond
		Backbone	CDR H1	A	N334	Side chain	H bond
		Side chain	CDR H1	A	K333	Side chain	H bond
B	Y32	Side chain	CDR H1	A	E341	Side chain	H bond
B	Y33	Side chain	CDR H1	A	D331	Side chain	H bond
B	H53	Side chain	CDR H2	A	D331	Side chain	Salt bridge
B	R97	Side chain	CDR H3	A	N338	Side chain	H bond
B	T99	Backbone	CDR H3	A	N334	Side chain	H bond
B	M101	Backbone	CDR H3	A	D331	Side chain	H bond
B	I102	Backbone	CDR H3	A	D331	Side chain	H bond
		Backbone	CDR H3	A	K212	Side chain	H bond
		Side chain	CDR H3				Hydrophobic
B	D107	Side chain	CDR H3	A	N338	Side chain	H bond
B	D184	Side chain	CDR L2	A	K212	Side chain	Salt Bridge
B	Y225	Side chain	CDR L3	A	K212	Side chain	H bond
				A	F209	Side chain	Hydrophobic
				A	I213	Side chain	Hydrophobic
				A	A216	Side chain	Hydrophobic
				A	I328	Side chain	Hydrophobic

† note that for the structure of PfRH5 bound to MAD8-151, the PDB is numbered according to the PfRH5ΔNL numbering, as the linker replacing the deleted loop is observed. The numbers given here are those for full-length PfRH5, to match the numbering in other structures, while the numbers in paratheses are those in the PDB, which match numbering for PfRH5ΔNL.
